# Cryptochrome proteins regulate the circadian intracellular behavior and localization of PER2 in mouse suprachiasmatic nucleus neurons

**DOI:** 10.1073/pnas.2113845119

**Published:** 2022-01-19

**Authors:** Nicola J. Smyllie, James Bagnall, Alex A. Koch, Dhevahi Niranjan, Lenka Polidarova, Johanna E. Chesham, Jason W. Chin, Carrie L. Partch, Andrew S. I. Loudon, Michael H. Hastings

**Affiliations:** ^a^Division of Neurobiology, Medical Research Council Laboratory of Molecular Biology, Cambridge CB2 0QH, United Kingdom;; ^b^Faculty of Biology, Medicine and Health, University of Manchester, Manchester M13 9PT, United Kingdom;; ^c^Division of Protein and Nucleic Acid Chemistry, Medical Research Council Laboratory of Molecular Biology, Cambridge CB2 0H, United Kingdom;; ^d^Department of Chemistry and Biochemistry, University of California, Santa Cruz, CA 96064

**Keywords:** CRY1, SCN, FRAP, nuclear retention, intracellular mobility

## Abstract

The ∼20,000 cells of the suprachiasmatic nucleus (SCN), the master circadian clock of the mammalian brain, coordinate subordinate cellular clocks across the organism, driving adaptive daily rhythms of physiology and behavior. The canonical model for SCN timekeeping pivots around transcriptional/translational feedback loops (TTFL) whereby PERIOD (PER) and CRYPTOCHROME (CRY) clock proteins associate and translocate to the nucleus to inhibit their own expression. The fundamental individual and interactive behaviors of PER and CRY in the SCN cellular environment and the mechanisms that regulate them are poorly understood. We therefore used confocal imaging to explore the behavior of endogenous PER2 in the SCN of PER2::Venus reporter mice, transduced with viral vectors expressing various forms of CRY1 and CRY2. In contrast to nuclear localization in wild-type SCN, in the absence of CRY proteins, PER2 was predominantly cytoplasmic and more mobile, as measured by fluorescence recovery after photobleaching. Virally expressed CRY1 or CRY2 relocalized PER2 to the nucleus, initiated SCN circadian rhythms, and determined their period. We used translational switching to control CRY1 cellular abundance and found that low levels of CRY1 resulted in minimal relocalization of PER2, but yet, remarkably, were sufficient to initiate and maintain circadian rhythmicity. Importantly, the C-terminal tail was necessary for CRY1 to localize PER2 to the nucleus and to initiate SCN rhythms. In CRY1-null SCN, CRY1Δtail opposed PER2 nuclear localization and correspondingly shortened SCN period. Through manipulation of CRY proteins, we have obtained insights into the spatiotemporal behaviors of PER and CRY sitting at the heart of the TTFL molecular mechanism.

## Significance

The suprachiasmatic nucleus (SCN), the master circadian clock of the mammalian brain, coordinates cellular clocks across the organism to regulate daily rhythms of physiology and behavior. SCN timekeeping pivots around transcriptional/translational feedback loops whereby PERIOD (PER) and CRYPTOCHROME (CRY) proteins associate and enter the nucleus to inhibit their own expression. The individual and interactive behaviors of PER and CRY and the mechanisms that regulate them are poorly understood. We combined fluorescence imaging of endogenous PER2 and viral vector–expressed CRY in SCN slices and show how CRYs, acting via their C terminus, control nuclear localization and mobility of PER2 to dose-dependently initiate SCN timekeeping and control its period. Our results reveal PER and CRY interactions central to the SCN clockwork.

Evolution has furnished organisms with biological clocks with periods of approximately one day (hence, circa-dian) that enable them to anticipate and thereby adapt to daily environmental cycles by temporally aligning their physiology and behavior (1). In humans, disruption of circadian control is a cause and/or a feature of metabolic, neurological, and psychiatric conditions (2, 3). In mammals, circadian rhythms are coordinated through a hierarchical system, where a master clock in the brain, the suprachiasmatic nucleus (SCN) consisting of ∼20,000 cells, synchronizes the cell-autonomous molecular clocks present in the majority of cells in the body (4). The widely accepted model for the molecular mechanism of the circadian clock is a delayed transcriptional–translational feedback loop (TTFL) whereby heterodimers of CLOCK:BMAL1 transactivate E-box–dependent transcription of *Period* (*Per1*, *Per2*) and *Cryptochrome* (*Cry1*, *Cry2*) genes (5). PER and CRY proteins subsequently accumulate and translocate from the cytoplasm, into the nucleus, to inhibit their own transcription. The stability of PER and CRY proteins is regulated through posttranslational modifications (6–8), such that their timely degradation alleviates repression and the cycle restarts on a ∼24-h basis. The TTFL of the SCN and peripheral tissues can be monitored by ex vivo real-time recording of circadian reporters, including the PER2::Luciferase knock-in fusion (9) and the *Cry1-Luciferase* transgene (10). In the absence of CRY1 and CRY2 (*Cry1^−/−^*, *Cry2^−/−^* [CryDKO]) or PER1 and PER2 (*Per1^−/−^*, *Per2^−/−^*), animals lose circadian behavior and ex vivo SCN and other tissues no longer oscillate (11–13).

Qualitatively, the model has therefore been useful, but we lack quantitative understanding of the clock mechanism and the behavior of its components, individually and interactively. This is especially true for the endogenous PER and CRY proteins, where biochemical studies from cell culture and tissue homogenates suggest they exist in dynamic, megadalton-scale macromolecular complexes (14–16). Importantly, the TTFL does not operate in isolation: clock proteins will undergo complex and dynamic spatiotemporal relationships as they navigate the crowded, compartmentalized environment of the cell. How the intracellular behaviors of clock proteins contribute to the cellular mechanism of the circadian clock is still poorly understood. Early studies characterized some mutual dependence for PER:CRY translocation to the nucleus (17–20), but they relied on the use of transgenes, overexpressed in cell lines, many of which lack a competent TTFL. They are, therefore, unlikely to capture accurately the endogenous intracellular behaviors and properties of the endogenous clock proteins. Therefore, we previously generated and validated the PER2::Venus (P2V) knock-in mouse, which enabled real-time imaging and quantification of endogenous PER2 in the SCN and peripheral cells (21). Critically, we discovered that PER2 nuclear localization is not subject to a temporal gate (a strong prediction from studies of *Drosophila* Per): rather, PER2 is predominantly nuclear across the entire circadian day, and it is remarkably mobile compared to other transcriptional regulators, not only within cellular compartments (nucleus or cytoplasm) but also between them. Moreover, we discovered that PER2 mobility is regulated by casein kinase 1, a determinant of SCN period. To expand understanding of clock protein behavior, we have now examined the close, collaborative relationship between PER2 and CRY proteins by combining imaging of P2V with a series of genetic manipulations of the CRY proteins, expressed via viral vectors.

## Results

### Cryptochromes Control Intracellular Spatiotemporal Behavior of PER2 in the SCN.

**Fig. 1.**
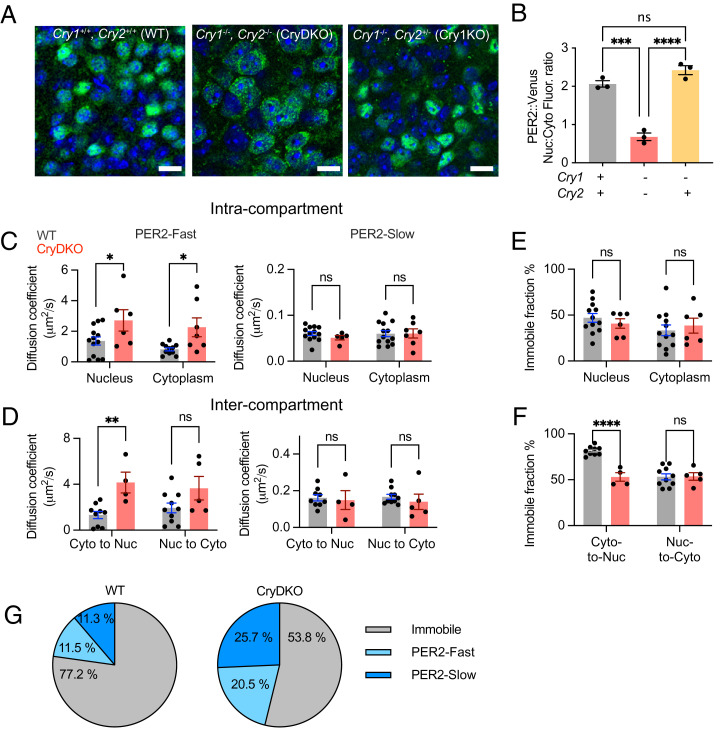
Cryptochromes control intracellular spatiotemporal behavior of PER2 in the SCN. (*A*) Representative confocal images showing intracellular localization of P2V (green), counterstained with DAPI (blue) in the SCN of adult WT, CryDKO, or Cry1KO mouse brain sections. (Scale bars, 20 μm.) (*B*) Ratio of nuclear (Nuc) to cytoplasmic (Cyto) P2V fluorescence in SCN, as in *A*. (*C*) Intracompartment diffusion coefficients, determined by FRAP, for PER2 in nucleus and cytoplasm of WT (gray) or CryDKO (red) organotypic SCN brain slices (*Left*: fast-moving, *Right*: slow-moving pools, as revealed by a two-component fit to recovery curves). (*D*) As in *C*, but comparing intercompartment mobility between nucleus and cytoplasm of P2V. (*E*) Proportion of P2V fluorescence identified by FRAP as immobile within compartments. (*F*) As in *E* for between-compartment mobility. (*G*) Proportions of P2V molecules in the three mobility categories in WT and CryDKO SCN slices. Statistical tests on the differences between sizes of these categories are included in *SI Appendix*. All group data are plotted as mean ± SEM. The dots represent individual SCN slices, which themselves are means of multiple single-cell measures. *P < 0.05; **P < 0.01; ***P < 0.001; ****P < 0.0001 assessed by one-way ANOVA.

We first assessed the intracellular localization of PER2 (reported as P2V) in adult mouse SCN sections. As expression approached its peak in late circadian day (21), P2V was predominantly nuclear in cells of wild-type (WT) SCN. In contrast, it was significantly more cytoplasmic in SCN of *Cry1, 2*–deficient (CryDKO) mice (Fig. 1*A*). Correspondingly, the nucleus:cytoplasm (nuc:cyto) ratio of P2V fluorescence in individual WT SCN cells was ∼2, (>1 indicates predominantly nuclear localization) but was <1 and significantly lower in CryDKO SCN (Fig. 1*B*). The presence of a single copy of *Cry2* was sufficient to sustain nuclear accumulation of P2V that was not significantly different from WT SCN (Fig. 1*B*). Importantly, this phenotype of increased cytoplasmic localization was observed for untagged WT PER2 protein, imaged through immunostaining (*SI Appendix*, Fig. S1 *A* and *B*), and thus not an artifact of the Venus tag. We next used fluorescence recovery after photobleaching (FRAP) in SCN organotypic slices to determine whether CRY proteins regulate PER2 mobility within and between nuclear and cytoplasmic compartments (Fig. 1 *C*–*G* and *SI Appendix*, Fig. S1 *C*–*G* and Videos S1–S4). Curve fits identified two components with distinct “fast-” or “slow-” moving pools of P2V (PER2-fast; PER2-slow), as defined by their relative diffusion coefficients. A proportion of P2V fluorescence did not recover over the course of the experiments, indicative of immobile or very slowly moving PER2 molecules (Fig. 1 *E* and *F* and *SI Appendix*, Fig. S1 *D* and *E*). As previously demonstrated (21), even though overall abundance changed, there was no circadian day/night difference in PER2 mobility in WT SCN (*SI Appendix*, Fig. S1 *D*–*F*), and so all WT measures were combined to compare mobility in WT and CryDKO SCN slices in a time-independent manner (Fig. 1 *C*–*G*). Strikingly, the mobility of PER2-fast was significantly increased in the absence of CRYs, within both nucleus and cytoplasm, as well as for movement into the nucleus. A similar trend for increased nucleus-to-cytoplasm mobility was not significant (Fig. 1 *C* and *D*). There were no such changes observed for PER2-slow within or between compartments (Fig. 1 *C* and *D*), and the absence of CRYs did not affect the proportion of immobile PER2 in nucleus or cytoplasm (Fig. 1*E*). In WT SCN slices subject to whole-nucleus bleaching, thus measuring cytoplasm-to-nucleus mobility, fluorescence recovery was low. Consistent with this, >77% of PER2 was immobile (Fig. 1*F*). In CryDKO SCN slices, however, the equivalent immobile pool was significantly smaller (<54%) than WT (Fig. 1*F*; *n* > 3 for each group), and consequently, mobile pools made up a greater proportion of PER2 than in WT slices (Fig. 1*G* and *SI Appendix*, Fig. S1*G*). In summary, in WT SCN, PER2 is predominantly nuclear and consists of three mobility pools: fast, slow, and immobile, the last being dominant. In the absence of CRYs, PER2 is located more in the cytoplasm than the nucleus and is, overall, more mobile in terms of both the mobility of individual molecules in the PER2-fast pool and the lower proportion of immobile PER2 molecules. Thus, the intracellular localization and mobility of endogenous PER2 in the SCN are modulated by CRY proteins, likely because by associating with CRYs, PER2 is incorporated into larger, more slowly moving complexes that facilitate its nuclear retention.

### AAV-Driven Cryptochrome Expression Reveals Differential Control over SCN Circadian Rhythms.

**Fig. 2.**
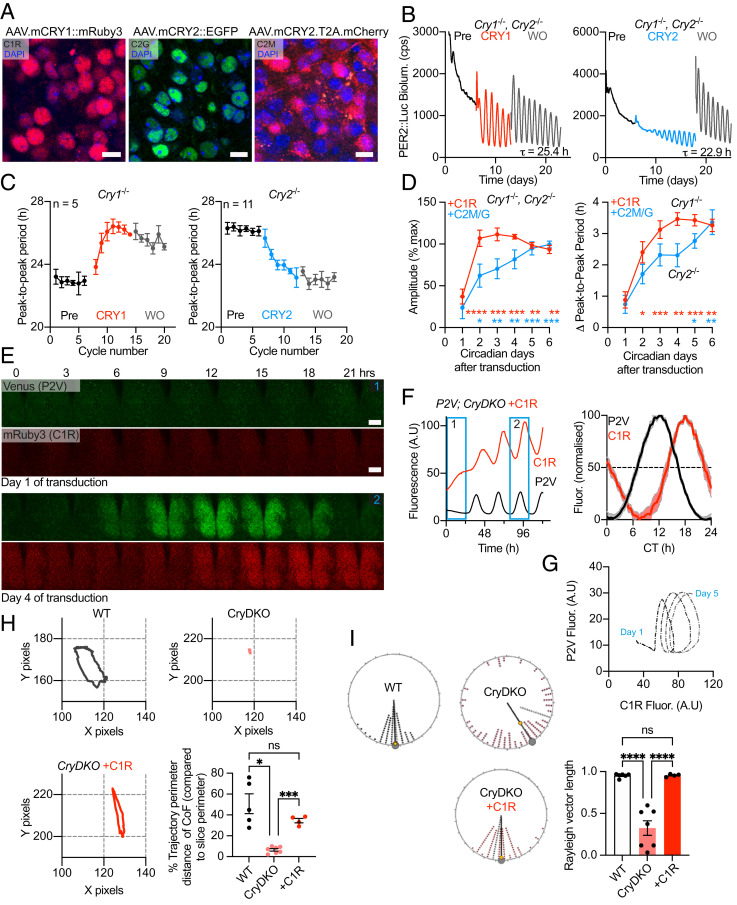
AAV-mediated CRY expression reveals differential control over SCN circadian rhythms and that it can direct the circadian spatiotemporal behavior of PER2. (*A*) Representative confocal images from organotypic CryDKO SCN slices of AAV-expressed CRY1 and CRY2, driven by respective minimal promoters and with mRuby (Cry1, *Left*) or EGFP (Cry2, *Center*) fusion tags, or free mCherry (Cry2, *Right*) (Scale bars, 10 μm.) (*B*) Representative PER2::Luc bioluminescence traces of CryDKO SCN slices transduced with either CRY1::mRuby3 (C1R, *Left*) or CRY2.T2A.mCherry (C2M, *Right*). Washout (WO) indicates change of medium, confirming stable rhythmicity after removal of excess AAV. (*C*) Circadian period, measured as peak-to-peak (P2P), of Cry1KO SCN slices transduced with C1R (*Left*) or Cry2KO SCN slices transduced with C2M or Cry2::EGFP (C2G) (*Right*, data combined). (*D*) Comparison of rate of effect (two-way ANOVA with Dunnett’s multiple comparison for testing significant difference compared with day 1 for each dataset: *P < 0.05; **P < 0.01; ***P < 0.001; ****P < 0.0001), following AAV transduction, of C1R (red) or C2M/C2G (blue, data combined). *Left*: Amplitude expressed as percentage of maximum for rhythms initiated in CryDKO slices, as in *B*. *Right*: Change in P2P period in Cry1KO (with C1R) or Cry2KO (with either C2M or C2G, data combined) SCN slices; as shown in *C*. (*E*) Montages of confocal images taken every 3 h, over 24 h, of P2V (green) and C1R (red) in a CryDKO SCN slice. *Upper* panels: First day after transduction with AAV-C1R. *Lower* panels: Fourth day after transduction, showing initiated rhythms of P2V and C1R fluorescence. (Scale bars, 200 μm.) (*F*) *Left*: Fluorescence intensity trace of the full recording shown in *E* (blue boxes indicate timing of images presented). *Right*: Mean 24-h profiles of P2V and AAV-expressed C1R across SCN slices (*n* = 6). CT registered to peak of P2V at CT 12. (*G*) Circular plot of temporal relationship between P2V and C1R expression evolving over the recording in *E* and *F*. On day 1 of transduction, both P2V and C1R fluorescence are low and arrhythmic. As C1R expression increases on day 2, oscillations begin for both proteins. A stereotypical, circular expression trajectory is instated in full by day 3. (*H*) Representative traces of center of fluorescence (CoF) analysis of P2V time-lapse recordings. *Top Left*: WT slices show a stereotypical spatiotemporal trajectory. *Top Right*: CryDKO slices do not show spatiotemporal organization. *Bottom Left*: AAV C1R initiates the spatiotemporal trajectory in CryDKO SCN. *Bottom Right*: Group data for trajectory perimeter distance (*P* > 0.05, ns; *P < 0.05; ***P < 0.001 assessed by Brown–Forsythe and Welch ANOVA). (*I*) *Left*: Representative Rayleigh plots (a measure of synchrony) showing peak phases of cell-like ROIs (each dot represents an ROI) in SCN slices. *Right*: Group data for Rayleigh vector length. Unless otherwise specified, all group data are plotted as mean ± SEM. *P* > 0.05, ns; **P < 0.01; ****P < 0.0001 assessed by one-way ANOVA.

To facilitate exploration of the mechanisms whereby CRYs modulate PER2 cellular behaviors and determine their consequences for SCN timekeeping, we designed adeno-associated viral vectors (AAVs) to express fluorescently tagged CRY1 (CRY1::mRuby3 or C1R) and CRY2 (CRY2::EGFP [enhanced green fluorescent protein] or C2G, and CRY2.T2A.mCherry or C2M), driven by minimal versions of their respective promoters (*SI Appendix*, Fig. S2*A*). Expression was confirmed through confocal imaging (Fig. 2*A* and *SI Appendix*, Fig. S2*B*) with transduction efficiency being >90% of cells, including almost all P2V-positive cells (*SI Appendix*, Fig. S2*C*; *n* > 3 SCN slices per group). In arrhythmic CryDKO SCN slices, all three AAVs initiated robust, high-amplitude rhythms of PER2::Luc (Fig. 2*B* and *SI Appendix*, Fig. S2 *D* and *E*). The C-terminal EGFP tag did not interfere with CRY2 circadian function and thus we pooled the data for the two CRY2 AAVs (C2G/M) (Fig. 2 *C* and *D* and *SI Appendix*, Fig. S2 *D*–*F*). The AAV-expressed CRYs were able to reset the period of SCN lacking one or other CRY: C1R expressed in *Cry1^−/−^* SCN lengthened period from ∼22 to ∼26 h, and C2G/M shortened period of *Cry2^−/−^* slices from ∼26 to ∼23 h (Fig. 2*C* and *SI Appendix*, Fig. S2 *G*–*I*; *n* > 4 per group). Finally, serial transduction of CryDKO SCN with C2G followed by C1R initiated rhythmicity and maintained a period of ∼25 h, confirming the appropriate interactions of the AAV-encoded CRY1 and CRY2 within the clock mechanism (*SI Appendix*, Fig. S2*J*). The slightly longer than WT period of 24 h is likely due to imprecise control of dose of each CRY protein, a point that we later address. In both CryDKO and single–Cry KO slices, the effects of AAV-CRY1 were more rapidly evident than AAV-CRY2 (Fig. 2*D* and *SI Appendix*, Fig. S2 *K* and *L*; *n* > 4 for each group). Nevertheless, although the changes in period effected by the two CRYs were comparable (*SI Appendix*, Fig. S2*M*; *n* > 4 for each group), AAV-CRY2-initiated SCN rhythms had higher amplitude compared to AAV-CRY1 (*SI Appendix*, Fig. S2*M*).

### Real-Time Fluorescence Imaging Shows That AAV-Driven CRY1 Can Direct the Circadian Spatiotemporal Behavior of PER2.

Having confirmed the efficacy of the CRY proteins, we next used confocal time-lapse imaging to visualize their behavior in relation to PER2. Only a few days after transduction of CryDKO slices, rhythmic C1R and C2G fluorescence signals were readily detected (Fig. 2*E* and *SI Appendix*, Fig. S3 *A* and *B*). Consistent with its more rapid circadian effects, C1R fluorescence increased faster than did C2G during the first 72 h after transduction (*n* = 3 per group) (*SI Appendix*, Fig. S3*C*), suggesting that the *pCry1* promoter strength is a significant contributor to the more rapid initiation of PER2::Luc rhythms by AAV-CRY1 (although we do not rule out additional effects of the respective proteins). We then employed dual real-time imaging to explore the cellular-level spatiotemporal relationship between PER2 and CRY1. Robust oscillations of C1R were detected across the SCN 1 to 2 d after transduction, and this was accompanied by rhythmic P2V fluorescence. Surprisingly, in the steady state established after 4 to 5 d, C1R peaked at ∼CT (circadian time)18, that is ∼6 h after the peak of P2V (Fig. 2 *E* and *F* and *SI Appendix*, Fig. S3 *D* and *E*), indicative of temporally distinct roles for PER2 and CRY1. We then visualized the evolving temporal organization between P2V and C1R in the SCN by plotting P2V fluorescence with respect to C1R fluorescence through time (Fig. 2*G*). Initially, fluorescence was low for both proteins, but AAV-CRY1 initiated strong P2V oscillations by the second day, and by the third day, C1R was also robustly circadian. The evolving early relationship between P2V and C1R presented a circular trajectory of growing amplitude, illustrating the phase difference between the two oscillations, where the peak of PER2 preceded that of CRY1. To incorporate spatial information, we then used region of interest (ROI) analyses (ROIs provide a proxy for single cells) to ask whether transduction by AAV-C1R was also able to initiate the network-level, spatiotemporal patterning of PER2, stereotypical of WT SCN (Fig. 2*H*). Before transduction, P2V signal in CryDKO SCN did not exhibit any spatiotemporal organization, nor cellular synchrony. Following transduction with C1R, P2V rhythms within individual cells across the SCN network became well organized in relation to each other, with a characteristic spatiotemporal wave of PER2 (Fig. 2*H*) and with high levels of synchrony (Fig. 2*I*), as observed previously for PER2::Luc bioluminescence (11). Furthermore, C1R itself also exhibited spatiotemporal organization, comparable to that of P2V, suggesting that the initiated wave of PER2 is a direct consequence of CRY1 expression and that PER2 and CRY1 influence each other at a network level (*SI Appendix*, Fig. S3 *F*–*H*). In summary, real-time imaging demonstrated, first, the efficacy of AAV-CRY1 and AAV-CRY2 vectors. Second, it showed that circadian expression of CRY1 is substantially phase delayed relative to PER2, and third, that expression of CRY1 establishes spatiotemporal organization of PER2 in previously disorganized, arrhythmic CryDKO SCN.

### AAV-Driven CRYs Can Relocate PER2 from the Cytoplasm to the Nucleus in CryDKO SCN.

**Fig. 3.**
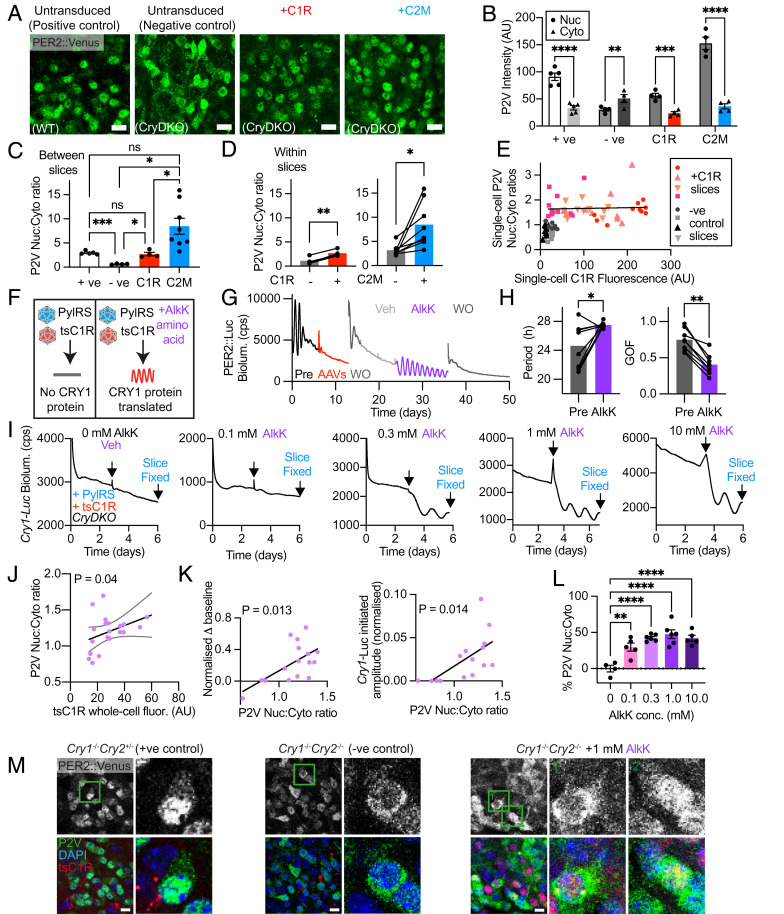
Dose-dependent control by cryptochromes of P2V behavior and SCN circadian function. (*A*) Representative confocal images showing localization of P2V (green) in organotypic SCN slices. From *Left* to *Right*, WT (+ve control), CryDKO (−ve control), CryDKO transduced with AAV-C1R, and CryDKO transduced with AAV-C2M. (Scale bars, 20 μm.) (*B*) Fluorescence intensity measures in the nucleus (Nuc) and cytoplasm (Cyto) of SCN cells, as in *A*. (*C*) P2V Nuc:Cyto ratio using raw data shown in *B*. (*D*) Within-slice comparisons (paired Student’s *t* test: *P < 0.05; **P < 0.01) of P2V Nuc:Cyto ratios of nontransduced (−) and transduced (+) cells in SCN treated with C1R or C2G. (*E*) P2V Nuc:Cyto ratio plotted against C1R fluorescence intensity across SCN conditions. Note transduction by C1R, regardless of expression level, results in complete restoration to WT state of PER2 localization (Pearson’s correlation: ns, *P* = 0.78). Each symbol is a single cell, ca. 10 cells/slice coded by symbol, four slices/group. (*F*) Schematic showing the design of the Amber ts system for dose-dependent control of C1R (tsC1R). (*G*) Representative PER2::Luc bioluminescence trace of CryDKO SCN showing reversible initiation of rhythmicity by expression of tsC1R triggered by noncanonical amino acid (AlkK). (*H*) Group data showing effect of AlkK-induced expression of tsC1R in (*Left*) initiation of stereotypical long-period (ca. 28 h) PER2::Luc rhythms with (*Right*) improved goodness-of-fit (GOF) (paired Student’s *t* test: *P < 0.05; **P < 0.01). (*I*) Representative *Cry1*-Luc bioluminescence traces of tsC1R-transduced CryDKO SCN slices treated with different concentrations of AlkK. A threshold of >0.1 mM was required for transcriptional inhibition and rhythm initiation. Recordings were terminated for confocal imaging three cycles after Veh/AlkK. (*J*) Correlation between nuclear localization of P2V and the level of tsC1R expression in CryDKO SCN as in *I* (*P* = 0.04, Pearson’s correlation). (*K*) Correlations between P2V Nuc:Cyto ratio and (*Left*) amplitude of the initiated *Cry1*-Luc rhythm and (*Right*) acute inhibition of *Cry1*-Luc. (*L*) Dose-dependent control of P2V Nuc:Cyto ratio by AlkK (data expressed as percentage of maximum, normalized to the mean +ve (100%) and −ve (0%) control values) using data shown in *SI Appendix*, Fig. S5*H*. Note that at the AlkK concentrations tested, P2V Nuc:Cyto does not reach WT levels (100%). (*M*) Representative confocal images of groups of cells with close-up images of single cells (green boxes in original image) from SCN slices fixed at *Cry1*-Luc peak expression. *Upper* panels show P2V as grayscale. *Lower* panels show P2V (green), tsC1R (red), and DAPI (blue). (Scale bars, 10 μm.) Unless otherwise specified, all group data are plotted as mean ± SEM. *P* > 0.05, ns; *P < 0.05; **P < 0.01; ****P < 0.0001 assessed by one-way ANOVA.

We next asked whether CRY1 and CRY2 could control the intracellular localization of PER2 in SCN slices. As observed in adult SCN brain sections (Fig. 1 *A* and *B*), P2V was predominantly nuclear in WT SCN slices (nuc:cyto ratio > 1) and cytoplasmic in CryDKO SCN slices (nuc:cyto ratio <1; Fig. 3 *A* and *B*). Transduction with either C1R or C2M relocated PER2 into the nucleus, leading to substantially higher P2V fluorescence intensity (Fig. 3 *A* and *B*). This was reflected by a significant increase in the nuc:cyto ratio of P2V, to levels not significantly different from those of WT slices (Fig. 3*C*). To test whether changes to P2V cellular localization were a cell-autonomous effect of AAV-CRY, we compared P2V nuc:cyto ratio between CRY-positive (CRY+) and rare CRY-negative (CRY−) cells within the same SCN slice, using mRuby3 or mCherry as markers for CRY expression (Fig. 3*D* and *SI Appendix*, Fig. S4). This showed that the P2V nuc:cyto ratio was significantly higher in CRY+ cells than in the few CRY− cells for both CRY1 and CRY2. Interestingly, C2M-transduced slices showed a trend of greater difference between nuc:cyto ratio of C2M+ cells and C2M− cells compared with the C1R+ and C1R− cells in C1R-transduced slices (Fig. 3*D* and *SI Appendix*, Fig. S4*C*). Measuring nuc:cyto ratios of the CRYs themselves showed that C2G was more nuclear than C1R (*SI Appendix*, Fig. S4*B*), thus mirroring the corresponding trends in P2V localization in the presence of AAV-CRY2.

### Control of P2V Behavior and SCN Circadian Function by Cryptochromes Is Dose-Dependent.

To acquire a more quantitative assessment of the effects of CRY proteins on PER2 intracellular behavior, we compared C1R fluorescence intensity and P2V nuc:cyto ratio on a single-cell basis. Across conditions, however, there was a binary rather than graded outcome. In the presence of AAV-CRY1, relocalization of PER2 to the nucleus appeared to be complete, regardless of the actual level of C1R fluorescence, suggesting that a threshold had been surpassed (Fig. 3*E*). To achieve finer, dose-dependent control of CRY1 at lower levels, we used a translational switching (ts) system, generating an AAV in which the C1R coding sequence contains an Amber stop codon substitution (*tsC1R*) (22) to make expression of AAV-CRY1 dependent on provision of the noncanonical amino acid, alkyne lysine (AlkK) (Fig. 3*F* and *SI Appendix*, Fig. S5 *A* and *B*). A transfer (t)RNA synthetase (with a blue fluorescent tag) capable of charging an orthogonal tRNA with AlkK was delivered in trans by a second AAV. We first confirmed that tsC1R fluorescence and initiation of PER2::Luc rhythms (in previously arrhythmic CryDKO SCN slices) were dependent on AlkK (Fig. 3 *G* and *H* and *SI Appendix*, Fig. S5 *B* and *C*). PER2::Luc rhythms were lost on removal of AlkK (Fig. 3*G*). As seen with nonswitchable C1R, the period of SCN rhythms initiated by tsC1R was ∼27 h, with comparable robustness (as assessed by goodness of fit) (*SI Appendix*, Fig. S5*D*). On application of increasing concentrations of AlkK, mRuby3 fluorescence increased accordingly in a dose-dependent manner (*SI Appendix*, Fig. S5*E*), although the intensity of fluorescence from tsC1R was lower than that of nonswitchable C1R (*SI Appendix*, Fig. S5*F*). Robust *Cry1*-Luc oscillations were initiated by AlkK at concentrations of 0.3 mM and above, whereas 0.1 mM AlkK was ineffective, thereby delineating a threshold between 0.1 and 0.3 mM (Fig. 3*I*). We found that this threshold represented ∼50% of endogenous CRY1 levels (at CT12) and approximately one-third of peak (CT18) endogenous CRY1 levels, as determined from a CRY1::mRuby3 knock-in mouse (23) (*SI Appendix*, Fig. S5 *G* and *H*). Correspondingly, there was a higher nuc:cyto ratio of tsC1R in cells treated with 0.3mM than with 0.1 mM (*SI Appendix*, Fig. S5*I*).

Having confirmed dose-dependent control of CRY1 expression and SCN function, we next asked whether the degree of PER2 relocalization was dose-dependent and, second, whether this correlated with dose-dependent control of SCN rhythms. Indeed, P2V nuc:cyto ratio significantly increased with AlkK dose (*SI Appendix*, Fig. S5*J*) and was correlated positively with the intensity of tsC1R fluorescence (Fig. 3*J* and *SI Appendix*, Fig. S5*K*). Moreover, this effect was cell autonomous, PER2 being localized to the nucleus of mRuby+ cells expressing tsCRY1 but not in the rare cells lacking tsCRY1 (*SI Appendix*, Fig. S5*L*). We next focused on the effects of tsCRY1 and PER2 localization on SCN rhythmicity. A characteristic feature of CRY-mediated initiation of SCN rhythms is an initial downward inflection that forms the first trough of the oscillation, resulting in a drop in *Cry1*-Luc baseline as transcriptional repression by newly translated CRY1 commences. This was observed at all effective concentrations of AlkK (Fig. 3*I*) and was positively correlated with the increased P2V nuc:cyto ratio (Fig. 3*K*). Equally, the amplitude of the tsCRY-induced rhythm was positively correlated with the degree of nuclear P2V. It should be noted, however, that relatively low levels of nuclear PER2 were sufficient to initiate and maintain clock function. At all effective concentrations of AlkK, the P2V nuc:cyto ratios were significantly lower than those of positive control (Cry2^+/−^) SCN slices, as well as slices initiated with nonswitchable C1R (Fig. 3*L* and *SI Appendix*, Fig. S5*J*). This was clearly evident, qualitatively, in the corresponding confocal images, where appreciable amounts of cytoplasmic P2V were observed in SCN with sustained rhythms (Fig. 3*M* and *SI Appendix*, Fig. S5*M*). We then compared the P2V nuc:cyto ratio with the nuc:cyto ratio of tsCRY1 at different AlkK concentrations. This revealed positive correlations at AlkK concentrations around the threshold of rhythm initiation (0.1 and 0.3 mM AlkK), consistent with a dose-dependent nuclear shuttling of PER2 by low levels of tsCRY1. At concentrations of AlkK at or above 1 mM, this relationship was lost, indicative of a saturation of the system (*SI Appendix*, Fig. S5*N*). Thus, by exploiting variable expression of tsCRY1, we have revealed a “tuneable” relationship between the degree of CRY-mediated nuclear localization of PER2 and SCN circadian timekeeping and the unexpected efficacy of relatively low levels of nuclear CRY1 and PER2 in sustaining rhythms.

### The CRY1 C-Terminal Domain Is Necessary for Effective SCN Pacemaking and Control over PER2 Nuclear Localization.

**Fig. 4.**
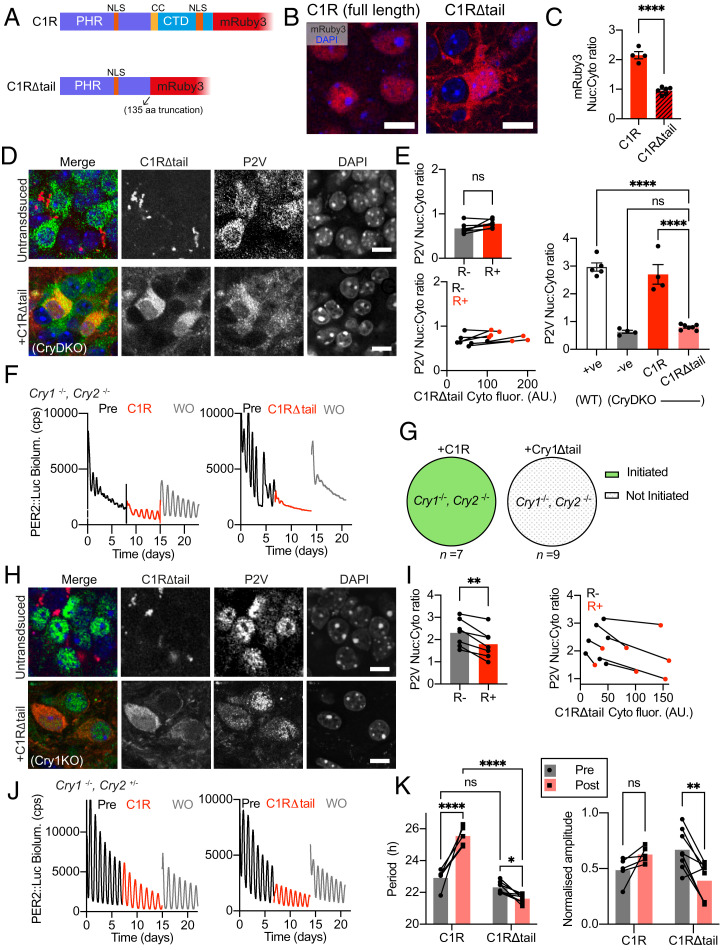
The CRY1 CTD is necessary for effective SCN pacemaking and control over PER2 nuclear localization. (*A*) Schematic showing the C1RΔtail fusion protein in which the final 135 amino acids of CRY1, including the coiled coil domain (CC) and all of the CTD with a nuclear localization signal (NLS), are deleted. (*B*) Representative confocal images of SCN cells in CryDKO slices transduced with (*Left*) full-length C1R, which has nuclear localization or (*Right*) truncated C1RΔtail, which has predominantly cytoplasmic localization. mRuby3 (red), DAPI (blue). (Scale bars, 10 μm.) (*C*) Comparison of mRuby3 Nuc:Cyto ratio measures in SCN slices expressing full-C1R or C1RΔtail (unpaired Student’s *t* test; *P* < 0.0001). (*D*) *Left*: Representative confocal images of SCN cells in CryDKO slices, untransduced (*Upper*), or transduced with C1RΔtail (*Lower*). (*E*) *Left*: P2V Nuc:Cyto ratios with paired analyses between untransduced (R−) and transduced (R+) cells within C1RΔtail-transduced SCN slices (paired Student’s *t* test). *Right*: P2V Nuc:Cyto ratios with between-slice comparisons of control and transduced slices (one-way ANOVA with Tukey’s multiple comparisons test). (*F*) Representative PER2::Luc bioluminescence traces of CryDKO SCN slices showing (*Left*) successful initiation of circadian rhythmicity by full-length C1R and (*Right*) unsuccessful initiation by C1RΔtail. (*G*) Pie charts showing success rates of initiation of PER2::Luc rhythms in CryDKO SCN slices. (*H* and *I*) as in *D* and *E*, respectively, but in Cry1KO SCN slices transduced with C1RΔtail. (*J*) Representative PER2::Luc bioluminescence traces of Cry1KO SCN slices transduced with (*Left*) C1R or (*Right*) C1RΔtail. (*K*) Group data (*Left*: circadian period; *Right*: normalized amplitude) showing paired analyses of Cry1KO SCN slices pre- or posttransduction with C1R or C1RΔtail (repeated-measures two-way ANOVA with Šídák's multiple comparisons test). Merged RGB images in *D* and *H* show P2V (green), C1RΔtail (red), and DAPI (blue) with separated grayscale channels shown alongside. (Scale bars, 10 μm.) Unless otherwise specified, all group data are plotted as mean ± SEM. The dots represent individual slices. *P* > 0.05, ns; *P < 0.05; **P < 0.01; ****P < 0.0001.

Having demonstrated the ability of CRY proteins to control PER behavior and SCN oscillation by using a gain-of-function approach, we then sought to test our model by exploring the effect of compromising the ability of CRY to enter the nucleus. To what extent does the nuclear localization of CRY1 influence PER2 behavior and SCN timekeeping? CRY1 has two predicted nuclear localization sequences (NLS): the first is well characterized, located in the Photolyase Homology Region (PHR); the second is poorly characterized and located in the C-terminal domain (CTD). In cell lines, deletion of a coiled-coil (CC) region in addition to the CTD caused complete relocalization of CRY1 to the cytoplasm and full exclusion from the nucleus (24). We therefore generated an AAV expressing C1R where the CC and CTD were deleted: CRY1Δtail::mRuby3 (C1RΔtail; Fig. 4*A*). We confirmed that the localization of C1RΔtail was indeed mostly cytoplasmic, where its nuc:cyto ratio was <1 in CryDKO and CRY1-deficient SCN slices, significantly lower than that of full-length C1R (Fig. 4*C* and *SI Appendix*, Fig. S6*A*). If CRY proteins associate with PER2 to affect its nuclear translocation, C1RΔtail should lose this capacity. Indeed, in the absence of any other CRYs in CryDKO SCN, transduction with C1RΔtail did not alter P2V localization (Fig. 4 *D* and *E*). It remained predominantly cytoplasmic, with nuc:cyto ratio < 1, and this ratio was not significantly different between mRuby3+ or mRuby3− cells within the same slices (Fig. 4*E*). In CryDKO SCN slices, transduction with full-length C1R initiated and sustained PER2::Luc rhythms (Fig. 4 *F* and *G*). In contrast, but consistent with C1RΔtail remaining in the cytoplasm and unable to drive PER2 into the nucleus, C1RΔtail failed to sustain or even initiate circadian rhythms in CryDKO SCN (Fig. 4 *F* and *G* and *SI Appendix*, Fig. S6*B*). These results highlight the importance of the CTD in the circadian function of CRY1 in the SCN and are consistent with C1RΔtail being a null allele. We then explored its effect in circadian-competent Cry1-null, Cry2^+/−^ SCN slices, in which PER2 is localized to the nucleus by CRY2 and which therefore oscillate with a stable but short circadian period. Transduction with C1RΔtail caused a modest but significant change in P2V localization, reducing levels in the nucleus and increasing cytoplasmic signal, with a consequent fall in its nuc:cyto ratio (Fig. 4 *H* and *I*). This effect was cell autonomous: evident in mRuby3+ cells but not mRuby3− cells (Fig. 4*I*). Moreover, the degree of P2V relocalization was dependent on the level of mRuby3+ expression (*SI Appendix*, Fig. S6*C*). Cells with higher mRuby3 fluorescence intensity tended to have less nuclear P2V compared those with lower mRuby3 signal and nontargeted cells from the same slice (*SI Appendix*, Fig. S6*D*). This was not reflected by corresponding increases in cytoplasmic P2V signal, perhaps indicative of greater degradation of PER2. Given the presence of WT CRY2 in these SCN, this suggests that C1RΔtail is not a null allele but rather displays dominant-negative behavior, attenuating CRY2-mediated nuclear localization of PER2. We therefore asked whether this altered mobility of PER2, as determined by CRY1Δtail, affected SCN circadian timekeeping. Cry1-null, Cry2^+/−^ SCN slices exhibited short-period (∼23 h) oscillations of PER2::Luc bioluminescence and, as previously shown, transduction with full-length C1R significantly lengthened period by ∼2.5 h, complementing the CRY1 deficiency but without affecting rhythm amplitude (Fig. 4 *J* and *K*). In contrast, C1RΔtail had two different effects on the SCN. First, it significantly shortened period by ∼1 h (Fig. 4*K*). Second, it reduced the amplitude of the rhythm of Cry1-null SCN slices by ∼40% (Fig. 4*K* and *SI Appendix*, Fig. S6*E*). Taken together, this suggests that the C-terminal tail is necessary for CRY1 to fulfill normal clock function, in part through nuclear entry for itself and for PER2 but that CRY1 can still interact with the clockwork, from the cytoplasm, in a way that is dominant to CRY2. In conclusion, our data show that CRY proteins are necessary for SCN circadian function through their role in regulating intracellular behavior of PER2, by ultimately promoting its nuclear retention.

## Discussion

By using live confocal imaging of P2V in WT SCN, we showed that endogenous PER2 is predominantly nuclear and present in three mobility pools: “fast,” “slow,” and “immobile.” Interestingly, PER2 exhibited similar nuclear mobility characteristics to BMAL1 in the SCN (25), which could reflect the incorporation of BMAL1 into PER-containing nuclear complexes. In CRY-deficient SCN, however, PER2 nuclear retention was compromised, resulting in more cytoplasmic localization. Importantly, we note that PER2 was not exclusively cytoplasmic: it is probable that PER2 can enter the nucleus in the absence of CRY proteins, likely regulated by its known NLS sequences (26), but requirement for CRY proteins lies in efficient nuclear retention. Intracellular mobility was also altered in the absence of CRYs. Specifically, in CRY-deficient SCN, the proportion of PER2 present in mobile pools was larger, with a corresponding reduction of immobile molecules. Importantly, the PER2-fast pool was more mobile in the absence of CRYs, but PER2-slow was not. We hypothesize that CRYs selectively reduce the mobility of particular classes of PER2-containing complexes but not those represented by PER2-slow. Instead, other components likely determine the overall mobility of this pool. Our findings support the idea that PERs and CRYs incorporate into several forms of multicomponent complexes rather than a unitary assembly (16), and these complexes exhibit different intracellular dynamics.

Confocal imaging of the C-terminal fluorescent tags confirmed that both AAV-CRY1 and CRY2 were expressed in a circadian manner under their respective minimal promoters. The tagged proteins were fully effective within the TTFL, insofar as they reprogrammed the period of single-mutant SCN and initiated circadian cycles of bioluminescence CryDKO SCN slices, with appropriate isoform-specific periods. Circadian initiation was faster with C1R, which we attribute to a stronger minimal promoter driving *Cry1* compared to *Cry2*. Indeed, the initial rate of production of C1R protein, inherently limited by transcription rate, was higher than for C2G. In addition, AAV-CRY1 and CRY2 driven by the same minimal *Cry1* promoter sequence show similar rates of initiation (11). At the network level, C1R initiated organized cellular rhythms of endogenous P2V in CryDKO SCN slices, accompanied by cellular synchrony and the establishment of a spatiotemporal wave comparable to WT SCN (11). C1R itself exhibited a spatiotemporal trajectory with the same magnitude as that of P2V. We hypothesize that the initiated PER2 wave was either directly attributable to the CRY1 wave, or that reciprocal interdependence was sufficient to organize their spatiotemporal patterning. Strikingly, we observed that the peak of C1R expression lagged that of PER2 by ∼6 h, peaking at ∼CT18. Although the mRuby3 tag has a slower folding time than Venus (27), this would not account for such a large phase difference. Recently, a similar phase relationship between PER2 and CRY1 was reported in CRISPR knock-in cell lines (28). This phase lag implies that PER2 and CRY1 will exert serial and not simultaneous actions within the TTFL. It also suggests that the composition of the negative complexes will evolve through the circadian cycle, including delayed CRY1-mediated transcriptional repression (29). Importantly, the late peak of CRY1 is followed in the SCN by a peak at ∼CT20 in the abundance of endogenous BMAL1 (imaged as Venus::BMAL1), a positive regulator in the TTFL (25). This temporal relationship will favor the progression from (poised) negative regulation by CRY1 to transcriptional activation by BMAL1:CLOCK heterodimers (5).

The initiation of PER2 rhythms by either CRY protein was accompanied by nuclear relocalization of P2V, with a trend for CRY2 to be more potent than CRY1. C2G itself had greater nuclear localization compared with C1R, which may underlie this enhanced PER2 nuclear signal. The cause of the difference in relocalization potency is unclear: it is unlikely to be through differences in the PHR domains of CRYs, which both bind the CRY binding domain (CBD) of PER2 with similar affinity (30). Instead, the secondary binding pocket may be a possible source: with its proposed role in differential period setting by CRY1 and CRY2 (31), it may contribute to PER2/CRY interactions. To investigate these interactions more quantitatively, we applied ts of CRY1 expression. We identified a threshold level of CRY1 (∼50% of CT12 endogenous CRY1 levels) that could initiate rhythms in CRY-deficient SCN, after which increasing CRY1 levels were progressively more effective on SCN rhythmicity (baseline and amplitude), consistent with previous reports (22). This was also associated with a dose-dependent increase in nuclear localization of PER2. By finetuning CRY1 at low levels across a relatively narrow window, we found that despite tsC1R initiating robust rhythms above its functional threshold, only ∼50% of endogenous PER2 was relocalized to the nucleus. Indeed, such efficiency is consistent with PER2 making high-affinity (∼28 nM) interactions with CRY1, thus able to maintain a highly effective PER:CRY-negative regulatory complex in the nucleus (32). We thus revealed an unanticipated feature of SCN timekeeping: relatively low levels of nuclear PER2 are sufficient to support robust SCN-wide rhythms. Furthermore, bringing together our measurements of PER2 intracellular behavior and CRY dosage, we propose that with increasing levels of CRY1 expression, more PER2:CRY1 heterodimers form, which in turn promote nuclear retention of PER2. This nuclear retention is necessary for PER:CRY to then act at E-box sites, which is central to TTFL pacemaking. The identified functional threshold therefore represents not only a critical level of CRY1 but also a critical level of nuclear PER2 (*SI Appendix*, Fig. S7). Importantly, the endogenous oscillation of CRY1 protein sits completely above the functional threshold (*SI Appendix*, Fig. S5 *G* and *H*). This ensures nuclear retention of PER2 across the entire day, and thus robust maintenance of SCN cellular timekeeping.

PER2 nuclear localization depended not only on the presence of CRY1 but also the ability of CRY1 to be retained in the nucleus. In the absence of other CRYs, expression of cytoplasm-localized C1RΔtail did not promote nuclear localization of P2V. Correspondingly, C1RΔtail did not initiate circadian function in CryDKO SCN. What was striking, however, was that in the presence of CRY2, which is normally sufficient to drive the TTFL, the C1RΔtail interfered with both SCN timekeeping and PER2 localization. The cytoplasmic C1RΔtail caused a proportion of PER2 to remain in the cytoplasm, reducing levels in the nucleus. This suggests that the remaining part of CRY1, notably the PHR domain, was able to interact with PER2, and/or other clock components, to retain it in the cytoplasm. Thus C1RΔtail was dominant over CRY2. Given that the PHR of CRY1, which remains in the C1RΔtail, binds to the PER2 CBD (30) as well as to the BMAL1 transactivation domain (30), the molecular basis for this dominant effect lies with other interactions. Functionally, this dominance was expressed as a shortening of the already short CRY2-dependent period. More rapid clearance of PER2 from the nucleus caused by C1RΔtail may be one source of this acceleration, as noted with destabilizing mutations of PER2 (33).

In summary: PER2 nuclear localization and molecular mobility depend on CRY proteins. By using AAV-expressed CRY variants, we have characterized their contribution to SCN-level spatiotemporal dynamics of PER2 and revealed marked phase delay of peak CRY1 to peak PER2. Translational switching enabled exploration of dose-dependent effects of CRY1 on PER2 and SCN rhythms. Surprisingly, the SCN clock cannot only tolerate but also produce robust oscillations when CRY1 levels are low and nuclear PER2 levels at only ∼50% of their normal distribution. Finally, CRY1 is dependent on its CTD to establish normal clock function by colocalizing to the nucleus with PER2. Taken together, we have revealed insights into the interdependence of PER2 and CRY proteins in the intracellular behaviors and wider SCN clock function.

## Materials and Methods

A detailed description of materials and methods are provided in *SI Appendix*. Itemized lists contain the provenance of all animals and previously generated AAVs used. For newly developed AAVs, the cloning steps to create the AAV constructs are described in detail. Other methods included are SCN organotypic slice preparation and all subsequent SCN recording procedures (confocal live/fixed imaging, FRAP, and luciferase recordings) as well as descriptions of analyses (FRAP, image based, and circadian). We also provide a full list of statistical tests for each figure.

## Supplementary Material

Supplementary File

Supplementary File

## Data Availability

All study data are included in the article and/or supporting information.
