# Supplementary material for: Cryptochrome proteins regulate the circadian intracellular behavior and localization of PER2 in mouse suprachiasmatic nucleus neurons
Source: Proc Natl Acad Sci U S A. 2022 Jan 19;119(4):e2113845119. doi: 10.1073/pnas.2113845119 (PMC8795536; doi:10.1073/pnas.2113845119)
Supplement: Supplementary File [file pnas.2113845119.sapp.pdf]

PNAS
www.pnas.org

Supplementary Information for

Cryptochrome proteins regulate the circadian intra-cellular behaviour and localisation of PER2 in mouse suprachiasmatic nucleus neurons

Nicola J. Smyllie[1*], James Bagnall[2], Alex A. Koch[2], Dhevahi Niranjan[1], Lenka Polidarova[1], Johanna E. Chesham[1], Jason W. Chin[1], Carrie L. Partch[3], Andrew S. I. Loudon[2] and Michael H. Hastings[1#]
Michael H. Hastings
Email: mha@mrc-lmb.cam.ac.uk

This PDF file includes:

Supplementary text
Figures S1 to S7
Figure legends for supplementary videos 1 to 4
SI References

Materials and Methods

Animals

All animal work was conducted under UK Home Office licence and overseen by the Animal Welfare and Ethical Review Body of the MRC Laboratory of Molecular Biology, under Animals (Scientific Procedures) Act of 1986. Animals were maintained on a 12:12 light-dark schedule.

Animal Provenance is set out in the table below (Table S1):

#	Strain name	Alleles	Provenance	Ref.
1	CryDKO (CRY1, CRY2-null)	$mCry1^{-/-}$; $mCry2^{-/-}$	Gijsbertus van der Horst, Erasmus Univ.	(1)
2	PER2::Luc	$mPer2^{Luc/Luc}$	Joseph Takahashi, UTSW	(2)
3	*Cry1*-Luc	*pCry1.Luc*	In house at MRC-LMB	(3)
4	PER2::Venus	$mPer2^{Venus/Venus}$	In house at University of Manchester	(4)
5	CryDKO x PER2::Luc	$mCry1^{-/-}$; $mCry2^{-/-}$; $mPer2^{Luc/Luc}$	Crossed 1 and 2 in house	N/A
6	PER2::Venus x CryDKO x *Cry1*-Luc	$mPer2^{Venus/Venus}$; $mCry1^{-/-}$; $mCry2^{-/-}$; *pCry1.Luc*	Crossed 1, 3 and 4 in house	N/A
7	CRY1::mRuby3	$mCry1^{mRuby3/mRuby3}$	In house at University of Manchester	(5)

AAVs

AAV design and production details are set out in the table below (Table S2):
Schematics of new AAVs are included in Figures S2A, S5A and 4A.

#	AAV	AAV full name	Construct provenance	AAV packaging
1	C1R	AAV.pCry1(min).mCry1.mRuby3	Modified in house from (6)	Penn Vector Core (University of Pennsylvania, USA)
2	C2G	AAV.pCry2(min).mCry2.EGFP		
3	C2M	AAV.pCry2(min).mCry2.T2A.mCherry		
4	PylRS	AAV.pSyn.TagBFP2.P2A.PylRS	In house (7)	
5	tsC1R	AAV1.pCry1(min).mCry1(177TAG).mRuby3	Modified in house from (8)	
6	C1RΔtail	AAV.pCry1(min).mCry1ΔCCtail.mRuby3	Modified in house from (6)	Vector Builder (USA)

The above AAV constructs were made as described below:
C1R was modified from AAV.pCry1.(min).mCry1.EGFP (6), where NcoI and BsrGI restriction sites were used to replace EGFP with mRuby3. mRuby3 was amplified from the mRuby-C1 plasmid (Addgene: plasmid #127808) by PCR using primers the following primers:

Forward: GTGAACCGTCAGATCCGCTA
Reverse: AGCGCGGCGATATCATCATC

C2G and C2M were modified from AAV.pCry1(min).mCry2.EGFP (6). The minimal pCry1 promoter was replaced with a minimal Cry2 promoter with the following sequence:

GAAAGGGCCCGGTAACAGGTCTGTCAGCCACCGGCCGGAGTCACCGCAACCCCTGTCT
TTCCCGGGGACGCGAAATGCGTAGGAGATCCGAGGTCCCTCCAGACTCAGCCCACGCG
GGGCGGACGCAGCGCGGTCCTGCGTCACCGGCGGGTGGCTACCAAGCGGGACCACAC
ATGGTGGGAACGTGAGGGGGCGGAGCCTGCGCCGACGCTGCTGACCAATGGAACAGA
GGGAGGGTCCCACCGGGTCTCACCCAATCACAGAGGCGATCCAGACTCCTTGGGGCG
GAGTCATGCCAGGGAGAGGCTGCATCATAGGTCGCTGGGCGGGCGCTGGGCGGGACC
AACGGGGCGGTCGGGCGGAGCGGCAGACCGAGACCCAGTCCAGGCGGCGGATATCAA
GATCTGGCCTCGGCGGCCAA

For C2M, as well replacing with the minimal pCry2 promoter, EGFP was replaced with T2A.mCherry.

tsC1R was modified from AAV1.pCry1(min).mCry1(177TAG).EGFP (8), where EGFP was replaced with mRuby3, by the same method as for the generation of C1R, as described above.

C1RΔtail modified from C1R, by amplifying only the first part of the *mCry1* coding sequence by PCR, leaving out the last 407 bp. MfeI and KpnI restriction sites were then used to replace the full-length coding sequence *mCry1* with the truncated version. The following PCR primers were used to introduce mfeI and KpnI restrictions sites into the 5’ and 3’ ends, respectively, of the PCR product:

Forward: AGGAATGGAACATCACTAAACTCTC
Reverse: GACACCGGTACCGTCATCGGTTTGGGGTAATTAACTC

The sequence of the deleted fragment is shown below:

GTGAACCATGCTGAGGCAAGCAGACTGAATATTGAAAGAATGAAGCAGATCTATCAGCA
GCTTTCCCGGTACAGAGGGCTAGGTCTTCTCGCCTCGGTCCCTTCTAACTCTAATGGGA
ATGGAGGGCTCATGGGCTATGCTCCTGGAGAGAATGTCCCGAGTTGTAGCAGCAGCGG
GAATGGAGGGCTCATGGGCTATGCTCCTGGAGAGAACGTCCCGAGCTGTAGCGGTGGA
AATTGCTCTCAAGGAAGTGGTATTTTACACTATGCTCACGGGGACAGTCAGCAGACTCA

CTCACTCAAGCAAGGGAGAAGCTCCGCGGGCACCGGCCTCAGCAGTGGGAAGCGTCCT AGTCAGGAAGAGGATGCCCAGAGTGTCGGCCCCAAAGTCCAGCGGCAGAGCAGTAAC

The above AAV plasmids were packaged into AAVs with serotype 1 backbone. All virus titres were at least $1x10^{13}$ GC/μL.

SCN fixed sections

Adult *Per2*$^{WT/WT}$ or *Per2*$^{Venus/Venus}$ mice carrying either *Cry1*$^{-/-}$,*Cry2*$^{-/-}$, or *Cry1*$^{-/-}$,*Cry2*$^{+/-}$ or *Cry1*$^{+/+}$,*Cry2*$^{+/+}$ combinations of *Cry* alleles (aged between 5-7 months old; both male and female animals; *n* =3 per group) were culled by a schedule 1 method (dislocation and exsanguination) at Zeitgeber Time (ZT) 9 and brains removed. Brains were post-fixed in 4% paraformaldehyde (Alpha Aesar) in 0.1M phosphate buffer for 4 hours at room temperature, with gentle shaking. The fixed brains were then cryopreserved in 20% sucrose in 0.1 M phosphate buffered saline (PBS) overnight at 4 °C. 40 μm coronal brains sections were prepared using a freezing microtome (Bright Instruments, U.K.), and washed in PBS for 5 minutes, 3 times. Sections containing the SCN were mounted on to frost-free slides (Thermo Fisher Scientific, USA) with a DAPI containing mounting medium (Vectashield Hardset with DAPI, Vector Labs, USA). For sections that were also immunostained, this was carried out as previously described (4), but described briefly below: free-floating sections were blocked for non-specific antiserum binding with 5% NGS (Vector Labs, USA) in PBS containing 1% BSA (Sigma, USA) and 0.3 % Triton X (Sigma, USA), referred to as PBS-BT. Sections were then incubated with rabbit anti-PER2 (made in house) at 1:500 in PBS-BT, shaking, for 16-20 hrs at 4°C. Sections were then washed twice with a 1:3 dilution of PBS-BT and incubated with goat anti-rabbit alexa 488 (Thermo Fisher Scientific, USA) for 1 h at room temperature. The sections were again washed twice in 1:3 PBS-BT, followed by 2 washes in PBS, before mounting as described above.

Organotypic SCN slice preparation

SCN slices were prepared as previously described (9) but also outlined briefly below: Both male and female mouse pups aged between P9-P12 were culled by a Schedule 1 method (dislocation and exsanguination) and brain removed, which was then quickly transferred to ice-cold GBSS (Sigma, USA) dissection medium (5 mg/mL glucose, 100 nM MK801 (Sigma, USA); 3mM $MgCl_2$ and 0.05 mM AP5, (Sigma); to block excitotoxicity). After trimming, 300 μm coronal brain slices were prepared on a McIlwain (U.K.) tissue chopper. The slice(s) containing the SCN was/were trimmed down further, minimising any extraneous non-SCN tissue. The SCN slices were transferred to membrane inserts (Milipore, USA) sitting on top of tissue culture medium (50% Eagle's basal medium (Sigma); 25% ESS (Sigma); 25% heat inactivated horse serum (Invitrogen, USA); 5 mg/mL D-glucose (Sigma); 25 μg/mL Penicillin/Streptomycin; 1% Glutamax (Invitrogen); pH7.2, osmolarity 315-320 mOsm), supplemented with 100 nM MK801

(Thermo Fisher Scientific, USA), 3mM $MgCl_2$ and 0.05 mM D-AP5 (Tocris, USA), to block excitotoxicity. After 2-4 hours, slices were transferred to fresh culture medium, without the excitotoxicity blockers, in 6-well dishes for long-term culturing (maintained at 37°C, 5% CO_2). SCN slices were cultured for 7 days prior to experiments involving luciferase recordings, or transferred directly to recording medium (see below) for experiments involving live fluorescence imaging.

SCN slice experimental procedures

SCN fixed slices

SCN slices were cut out of their membrane inserts and fixed in 4% PFA in phosphate buffer (as previously) for 30 minutes at room temperature, with gentle shaking. Fixed SCN slices were washed with PBS for 15 minutes, 3 times and mounted on to slides (as previously) with mounting medium either with or without DAPI depending on fluorophores present in the slices (i.e. no DAPI for those containing tagBFP2).

Luciferase recordings

SCN slices were transferred to 35 mm dishes containing 1.2 mL recording medium (D-MEM (Sigma); 0.35 mg/mL $NaHCO_3$ (Fisher Scientific); 5 mg/mL glucose; 25 µg/mL Penicillin/Streptomycin; 0.01M HEPES (Invitrogen)) made up as a stock solution and further supplemented with foetal calf serum (Gibco, USA), B27 (Gibco), Glutamax (Invitrogen) and 10 µM Luciferin (Microsynth, Switzerland)). Dishes were sealed with a cover-glass secured using silicone grease to prevent evaporation of media during the recording. Luciferase bioluminescence was detected by a photon multiplier tube (PMT; Hamamatsu, Japan), maintained at 37 °C in a light-tight incubator (CO_2 not required as slices were sealed and buffered with HEPES and $NaHCO_3$). Photon counts were recorded every second and counts were combined in 6-minute bins.

Viral transduction of SCN slices

SCN slices were transduced with AAVs after a medium change (culture medium or recording medium, depending on the experiment) immediately prior to transduction. One µL of AAV with titre of at least $1x10^{13}$ GC/mL was dispensed directly on top of the SCN slice and incubated for 7 days before exchanging for fresh medium.

Initiation of Cry expression in *Cry1/2* deficient slices

CryDKO SCN slices were assessed for arrhythmic phenotype using PER2::Luc or *Cry1*-Luc PMT recordings for at least 4 days, or longer in cases where residual short period and unstable oscillations persisted (a known phenotype for Cry deficient tissues). Slices where arrhythmicity was confirmed were transduced with the CRY AAVs. PMT recordings incorporated the baseline phenotyping phase, 7 days transduction phase and at least 7 days after washout of

the AAV, the last phase being when expression is stable. “Pre” and “AAV” comparisons were made using the phenotyping phase and the washout phases of the recordings.

For assessing the effect of Cry initiation on PER2::Venus localisation, *Cry1*-Luc PMT recordings were made as above, but the SCN slices were fixed (as described previously) between 2-4 days after the final medium change, at the CT12 as defined by *Cry1*-Luc rhythms (peak at CT13 (10)), and imaged by confocal (described later).

Single Cry KO SCN slices (either *Cry1*$^{-/-}$ or *Cry2*$^{-/-}$) were put through a similar experimental procedure, the only difference being in the baseline phenotyping phase. Here, the baseline recording was for at least 5-7 days before transduction, to allow for “peak-to-peak” period determination. For dual initiation of both *Cry1* and *Cry2* expression, the slices were transduced first with CRY2-T2A-mCherry AAV for 7 days, followed by a medium change and then subsequently transduced with CRY1::mRuby3, followed by a final medium change, where again, 7 days of recording allowed for a final period determination.

Translational switching

As with non-ts dependent initiation of *Cry* expression, CryDKO SCN slices were assessed for arrhythmic phenotype using either PER2::Luc or *Cry1*-Luc reporters. SCN slices were then transduced simultaneously by application of both AAVs: PylRS and tsC1R (1 µL of each AAV). For the validation experiments, the SCN slices were incubated with AAV for 7 days prior to medium change with fresh recording medium. After at least 2 days of further PMT recording to confirm baseline arrhythmic phenotype, the substrate for PylRS, alkene lysine, abbreviated to AlkK, (*N6-{(2-propynyloxy)carbonyl}-l-lysine* (synthesised in-house) stored as 100 mM stock solution made in recording medium, adjusted to pH 7.0) was added to the recording medium with a final concentration of 1 mM. Reversibility was confirmed by exchanging fresh recording medium that did not contain Alk, using four washouts for 15 minutes each. In the dose response experiments, AlkK was applied to give a final concentration of either 0, 0.1, 0.3 1 or 10 mM to the double transduced P2V; CryDKO SCN slices. After confirmation of initiated circadian rhythmicity, at 2-4 days after AlkK application, SCN slices were fixed at CT12, reported by *Cry1*-Luc, and P2V was imaged by confocal.

Confocal time-lapse recordings

Live imaging of SCN organotypic slices was carried out using either Zeiss LSM780 or LSM880 inverted confocal systems, maintained at 37°C. Custom inserts were made to hold up to 6x 35 mm dishes at a time. The “position list” function was used to make simultaneous time-lapse recordings from multiple SCN slices. SCN slices were transferred to 35 mm glass bottom dishes (Mattek) for all live imaging experiments. The same recording medium was used as for the PMT luciferase recordings. For time-lapse recordings, a 10X apochromatic objective was

used with the following acquisition parameters: 1024 x 1024 pixel frame size, 4x averaging, 1 frame acquired every 30 minutes for at least 60 hours.

Confocal FRAP

FRAP experiments were carried out in SCN organotypic slices, as previously described (4), using a Zeiss LSM780 inverted confocal, with a 63x apochromatic objective, with the whole system maintained at 37 °C. SCN slices were cut out of their membrane inserts and inverted so that the tissue had direct contact with the glass bottom dish. Excess recording medium was removed from the dish (leaving ~100 µL remaining). The slice was held in place using a slice harp (Scientifica, U.K.), to enable stable imaging. To reduce photobleaching, only the regions of interest (ROIs) were imaged, rather than full-frame imaging. FRAP of the Venus signal was carried out using the following protocol: 20 frames baseline acquisition, 10 frames photobleach at 100% intensity using the 514 laser, 100 seconds of acquisition to capture fluorescence recovery (2 fps). Alongside the FRAP ROI, a background ROI and a control, unbleached cell ROI were also recorded from to account for background fluorescence and acquisition photobleaching. Analysis of recovery curves was conducted in the Zen acquisition software (Zeiss, Germany). Here, background subtraction, acquisition bleach correction and normalisation were automatically applied before the FRAP curve fitting procedure. Recovery data were then fit to the best fit model, a two-component, passive diffusion fit:

$$I = I_E - I_1 exp^{-\frac{t}{T_1}} - I_2 exp^{-\frac{t}{T_2}}$$

I =Intensity at time, t

I_E = End intensity (after recovery)

$I_{1,2}$ =Intensity from component 1 or 2

$T_{1,2}$ =Constant for component 1 or 2

Zen generated outputs of $t_{1/2}$ and % for each mobility component. I_1 was automatically assigned to the fastest moving component, thus we termed this $PER2_{fast}$ and I_2 as $PER2_{slow}$.

$T_{1/2}$ values were calculated from the curve fit using the following equation:

$$t_{1/2} = ln2 \cdot T_x$$

(where T_x is either T_1 or T_2 for $PER2_{fast}$ or $PER2_{slow}$ respectively)

The $t_{1/2}$ values were then converted to diffusion coefficients using the Axelrod equation (11), which accounts for different sizes of bleach area:

$$D = \frac{w^2}{4 \cdot t_{1/2}}$$

D = diffusion coefficient

w = bleach diameter

For each experiment, % mobile PER2 molecules is given by the magnitude of fluorescence recovery ($I_E - I_0$) as a percentage of the initial pre-bleach fluorescence ($I_I - I_0$).

$$\% \, PER2_{mobile} = \left(\frac{I_E - I_0}{I_I - I_0} \right) * 100$$

I_I =Initial intensity (before FRAP)

I_0 =Intensity at $t = 0$ after photobleaching.

Percentage of each fraction is given by the following equations:

Mobile fractions: $\% \, PER2_{Fast} = \left(\frac{I_1}{I_E - I_1 - I_2}\right) * 100$

$\% \, PER2_{Slow} = \left(\frac{I_2}{I_E - I_1 - I_2}\right) * 100$

Immobile fraction: $\% \, PER2_{immobile} = (100 - (\% \, PER2_{Fast} + \% \, PER2_{Slow})$

FRAP ROIs were either “spot bleaches” within either cytoplasm or nucleus, where each ROI circle was 2 µm in diameter, or whole “compartment bleaches” where the entire cytoplasm or nucleus was targeted. In rhythmic (WT) SCN slices FRAP experiments were carried out at CT2 and CT12, as defined by PMT recordings of *Cry1*-Luc reporter. For each SCN slice, 12 different cells were put through the FRAP protocol, 3 cells for each type of ROI (cytoplasm/nucleus spots, or cytoplasm/nucleus whole compartment). This relatively low n number for cells/slice is due to the amount of time required to conduct each FRAP experiment whilst remaining within one hour of the circadian time point. At least 6 SCN slices were used for each time point. In CryDKO SCN slices CT was not definable.

Confocal snapshot imaging

Fixed SCN tissue (sections and slices) were imaged using either Zeiss LSM710, 780 or 880 systems using a 63x oil immersion apochromatic objective. To image the whole coronal view of the SCN, a tile-scan protocol was used within the Zen acquisition software. Particularly in the DAPI channel, this produced some tile artifacts at the joins between tiles, which can be seen in images. Nonetheless, the tile-scan allowed for higher resolution images of the whole SCN than could be acquired with the highest numerical aperture (NA) 10x objective, an objective that would not have required tiling. Further processing was carried out within FIJI (12).

Analysis methods

Circadian rhythm analysis

SCN circadian rhythmicity was assessed through whole-slice emission of luciferase bioluminescence, as measured by PMTs (as described above) or by mean average slice fluorescence (average fluorescence intensity of the slice across each frame). In SCN slices with multiple fluorescence reporters, the fluorescence rhythms could be compared with one another.

BioDare

The Fast Fourier Transform – Linear Non-Least Squares (FFT-NLLS) within the BioDare (www.biodare2.ed.ac.uk) circadian rhythms analysis software package (13) was used to analyse rhythmicity of both luciferase and fluorescence-based recordings, where there was stable rhythmicity of at least 5 days. The first 24 hours after any medium change or treatment were excluded from the analysis. The output from software included best-fit period within a circadian-relevant window of 18-32 h periods, relative peak phase, amplitude, goodness of fit (GOF) and relative amplitude error (RAE), the latter are an assessment of the robustness of the rhythms.

Peak-to-Peak period and delta changes

Peak-to-Peak period was calculated to assess changes of period on a cycle-by-cycle basis, and was carried out within Graphpad Prism (v8 and v9, Graphpad Software). Raw data were first de-trended using a centred fifth exponential fit (unbiased detrending). The "area under the curve" analysis was then used to define peaks that were more than 10% above the baseline (baseline = 0 after detrending). The time between peaks was calculated and plotted as peak-to-peak period. The peak-to-peak Δ was calculated as the difference between consecutive peak-to-peak period intervals. To account for the inter-slice variability in absolute period change across a recording, the peak-to-peak Δ was expressed as a percentage of the maximum period change across the experiment.

Circadian rhythm amplitude and normalisation

In CryDKO SCN slices transduced with Cry AAVs, circadian rhythm amplitude was used as a measure of the robustness of the newly initiated rhythms. Amplitudes of PER2::Luc oscillations were determined on a peak-to-peak basis using the "area under the curve" feature in Graphpad Prism, where peaks were defined as described above. Circadian rhythm amplitude is known to be variable between SCN slices, so peak-to-peak amplitude was normalised to the maximum amplitude during the stable "washout" phase (after the final medium change) of the recording.

Average circadian profile generation and phase mapping of C1R

The average peak phase of C1R was mapped to circadian time by comparing to the peak P2V, which was defined as CT12 (4). Peak-to-Peak intervals were defined for each reporter in Graphpad Prism (Graphpad), as described above, for at least 4 cycles. The individual peak times, expressed in circadian time, were then used to calculate the mean average CT for the peak of C1R for each slice (N =6). Once the mean peak time for C1R was calculated, this was then used to generate average 24-hour circadian profiles for both P2V and C1R. These profiles were generated from a single circadian day (recordings scaled to 24 hours), where the circadian peaks of fluorescence were normalised to 100% for each slice. The peak times were then aligned to CT12 and CT18 for PER2 and CRY1, respectively, across all slices. After peak

alignment, each circadian time point (0.5 circadian hours) was averaged across all slices (N =6) to generate the average profile.

Initiation dynamics of AAV-expressed Cry proteins

Confocal time-lapse recordings (as described above) were made at the point of AAV transduction to directly track protein initiation dynamics. A simple linear regression was fit over the first 72 hours of each recording to calculate the slope. This slope was used as an estimate of initial rate of protein production.

Imaging analyses

ROI and spatio-temporal analysis of time-lapse recordings

The Semi-automated route for image analysis (SARFIA) (14) package within Igor Pro (Wavemetrics, USA) was used for spatio-temporal analyses of time-lapse recordings. Prior to analyses, the raw recordings were processed by removing imaging artifacts (despeckling) and background subtraction from an ROI (outside of the slice) using FIJI (NIH, USA).

For ROI-based analyses, SARFIA was used to determine cell-like ROIs using thresholding and Laplacian edge detection. From this, time-lapse series were generated for each ROI and subsequently analysed using BioDare to assess circadian properties, as described above. Rhythmic synchrony/coherence of the slices were assessed using the relative peak phase of each ROI (output from BioDare) and applying a Rayleigh analysis using bespoke software (https://github.com/tomoinn/web-statistics). Rayleigh analysis outputs included vector length and a circular plot with peak times of the individual ROIs.

For spatio-temporal analyses, an in-house script was run in the SARFIA package of Igor Pro, which generated a “Centre-of-Fluorescence” (CoF) xy coordinate time-series for each slice. The xy series for one circadian day for each slice was plotted in Prism (Graphpad, USA) to generate a daily trajectory across the slice. Larger, directional deviations correspond to greater spatio-temporal organisation, whereas small or noisy trajectories correspond to less spatio-temporal organisation.

Intracellular fluorescence measurements and Nuclear:Cytoplasmic ratio calculation

Fluorescence intensity measures were determined manually using FIJI (NIH, USA) in both fixed SCN brain sections from adult mice and fixed SCN brain slices from neonatal pups (as previously described). All measures were background-corrected using fluorescence intensity located outside the slice area. ROIs for were selected for whole-cell, nucleus or cytoplasm.

The average intensity within ROIs in the nucleus, were delineated by DAPI nuclear staining, and cytoplasm (outside DAPI+ area of cell) were measured. The Nuc:Cyto ratio was then calculated using the background-subtracted intensities and expressed as percentages. For fixed SCN sections, Nuc:Cyto was determined for 10 cells within each SCN section and at least *n* =3 SCN sections per mouse, with a total of *n* =3 mice per genotype group. For fixed SCN slices, the ratio was determined for at least 10 cells for each slice, where in transduced slices 10 CRY+ and 10 CRY- cells were sampled for each slice, defined by AAV-dependent fluorescence (mRuby3 or mCherry). Fluorescence intensity measurements were made for P2V and C1R. *n* >3 SCN slices were used for each group.

Statistics

Other than the Rayleigh analysis (described above), all graphs and statistics were performed in Prism (V8, V9, Graphpad). Graphs showing group data have error bars expressing standard error of the mean (SEM). The table below outlines the statistical tests and *n* numbers for each figure:

Figure	# groups	# n per group	Test	Comparison
1B	3	3 brain sections/ animal; *n* =3 animals/group	One-way ANOVA	Tukey’s
1C-E	2 * 2	*n* >4 slices/group	Two-way ANOVA	Tukey’s
S1B	2	*n* ≥3 brain sections/ animal; *n* ≥3 animals/ group		
S1C	4 * 2	*n* >4 slices/group	Two-way ANOVA	N/A
S1D-E	2	*n* >4 slices/group	Unpaired Student’s t-test	N/A
2D	6 * 2	*n* >5 slices/group	Mixed Effects Model	Dunnett’s T3
2H	3	*n* ≥3 slices/group	Brown-Forsyth and Welch ANOVA	Dunnett’s T3
2I	3	*n* ≥4 slices/group	One-way ANOVA	Tukey’s
S2D, F, H, I	2	*n* >4 slices/group	(Un)paired Student’s t-test	N/A
S2M	2 * 2	*n* >4 slices/group	Two-way ANOVA	Šídák’s
3B	2 * 4	*n* ≥4 slices/group	Two-way ANOVA	Šídák’s
3C	4	*n* ≥4 slices/group	Brown-Forsyth and Welch ANOVA	Dunnett’s T3
3D	2	*n* ≥4 slices/group	Paired t-test	N/A
3E	N/A	*N* =10 cells /slice; *n* =4 slices/group	Pearson’s correlation	N/A
3H	2	*n* =6 slices	Paired Student’s t-test	N/A
3J	N/A	*n* =20 slices	Pearson’s correlation	N/A

3K	N/A	*n* =16 slices	Pearson’s correlation	N/A
3L	5	*n* >4 slices/group	One-way ANOVA	Holm-Šídák’s
S3C	2	*n* ≥3 slices/group	Unpaired Student’s t-test	N/A
S4B	2	*n* =4 slices/group	Unpaired Student’s T-test	N/A
S5D	2	*n* >5 slices/group	Unpaired Student’s t-test	N/A
S5E	5	*n* ≥4 slices/group	One-way ANOVA	Dunnett’s T3
S5F	5	*n* >5 slices/group	One-way ANOVA	Tukey’s
S5H	4	*n* ≥4 slices/group	One-way ANOVA	Tukey’s
S5I	2	*N* ≥49 cells/group pooled from *n* =5 slices/group	Unpaired Student’s t-test	N/A
S5J	7	*n* ≥4 slices/group	One-way ANOVA	Tukey’s
S5K	N/A	*N* =220 cells pooled from *n* =27 slices	Pearson’s Correlation	N/A
S5N	N/A	*N* ≥ 40 cells/group pooled from *n* =5 slices/group	Pearson’s Correlation	N/A
4C	2	*n* ≥4 slices/group	Paired Student’s T-test	N/A
4E left	2	*n* = 7 slices	Paired Student’s T-test	N/A
4E right	4	*n* ≥4 slices/group	One-way ANOVA	Tukey’s
4I	2	*n* = 6 slices	Paired Student’s T-test	N/A
4K	2*2	*n* >4 slices/group	Two-way ANOVA	Šídák’s
S6A	2*2	*n* ≥4 slices/group	Two-way ANOVA	Tukey’s

SI Figures

Fig S1

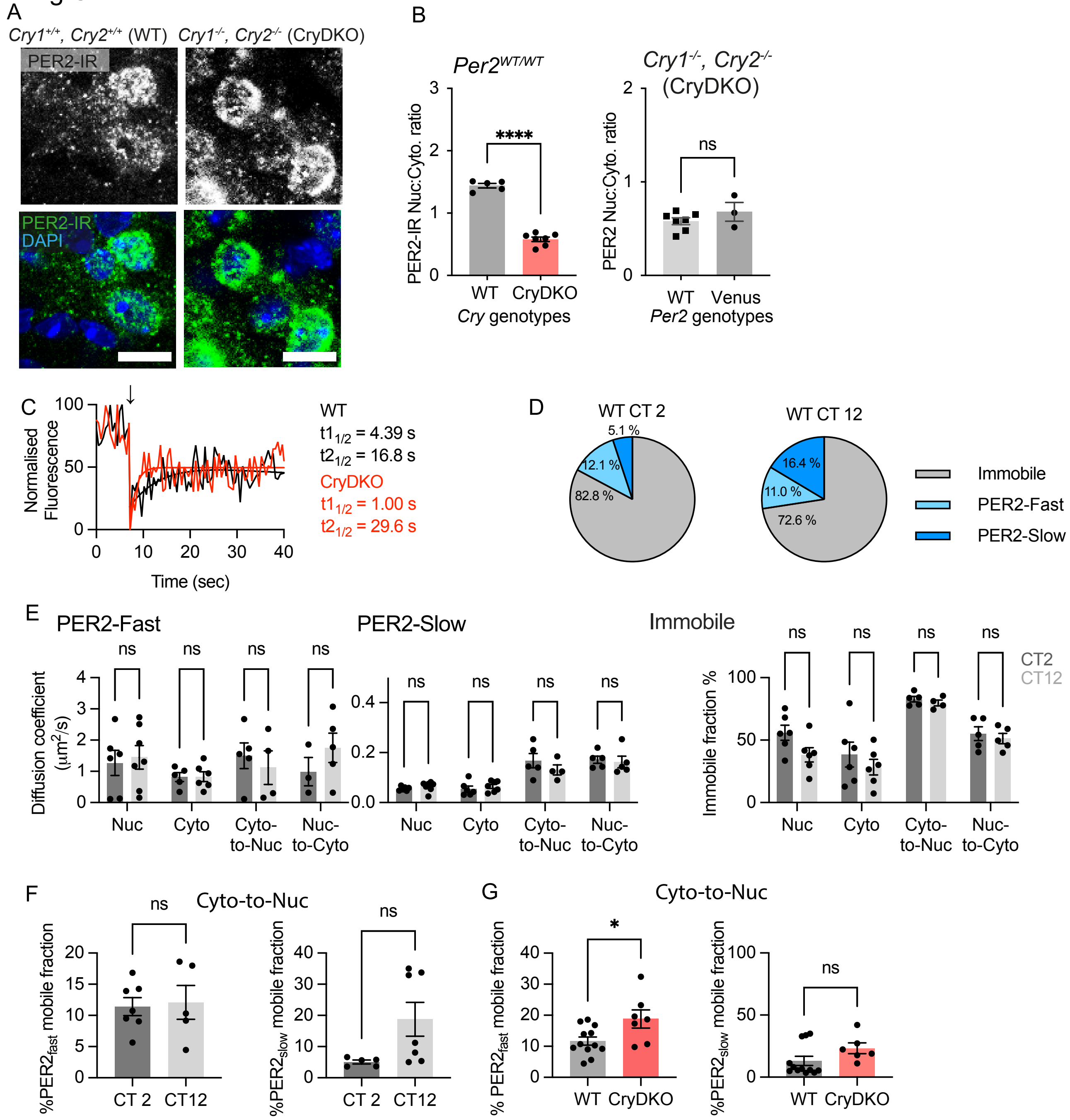

Figure S1. PER2 localisation and mobility in SCN slices. (A) PER2::Venus shows the same localisation as WT PER2 (without the Venus tag). Representative confocal images of PER2 immunofluorescence (PER2-IR) of WT PER2, in adult SCN sections (ZT9). Scale bar = 10 µm. (B) Group data showing nucleus:cytoplasm (nuc:cyto) ratio for either (left) PER2-IR fluorescence in CRY WT and CryDKO SCN or comparing (right) PER2-IR fluorescence and P2V native fluorescence in CRY WT SCN, all from adult brain sections (ZT9). There is no significant difference between PER2 nuc:cyto ratio of CryDKO SCN whether measured for WT PER2 or P2V. (C) Representative FRAP curves for WT and CryDKO SCN slices. Arrow indicates when photobleach was applied. $T1_{1/2}$ and $T2_{1/2}$ represent the half-time of fluorescence recovery for the fast and slow components, respectively. (D) Proportions of P2V molecules in the 3 mobility categories (PER2-Fast, PER2-Slow and Immobile) in WT SCN slices at CT2 and CT12. (E) Group data showing FRAP-derived P2V diffusion coefficients of (left) PER2-Fast and (middle) PER2-Slow pools of PER2 from WT SCN slices recorded from at either CT2 or CT12, using *Cry1*-Luc bioluminescence to determine circadian phase. Right: Group data for the percentage of immobile/very slow PER2 molecules ($p > 0.05$ ns; assessed by two-way ANOVA, where compartment and time are the two variables). (F) Group data showing percentages of (left) PER2-fast and (right) PER2-slow fraction sizes in WT slices measured at CT2 and CT12. (G) as in (F), but comparing pooled WT (CT2 and 12) and CryDKO slices ($p > 0.05$ ns; $p < 0.05$ *; assessed by unpaired t-test). Group data are plotted as mean ± SEM. The dots represent individual SCN slices, which themselves are means of measures from multiple SCN sections (in B) or single cells (in E-G). Related to Figure 1.

Fig S2

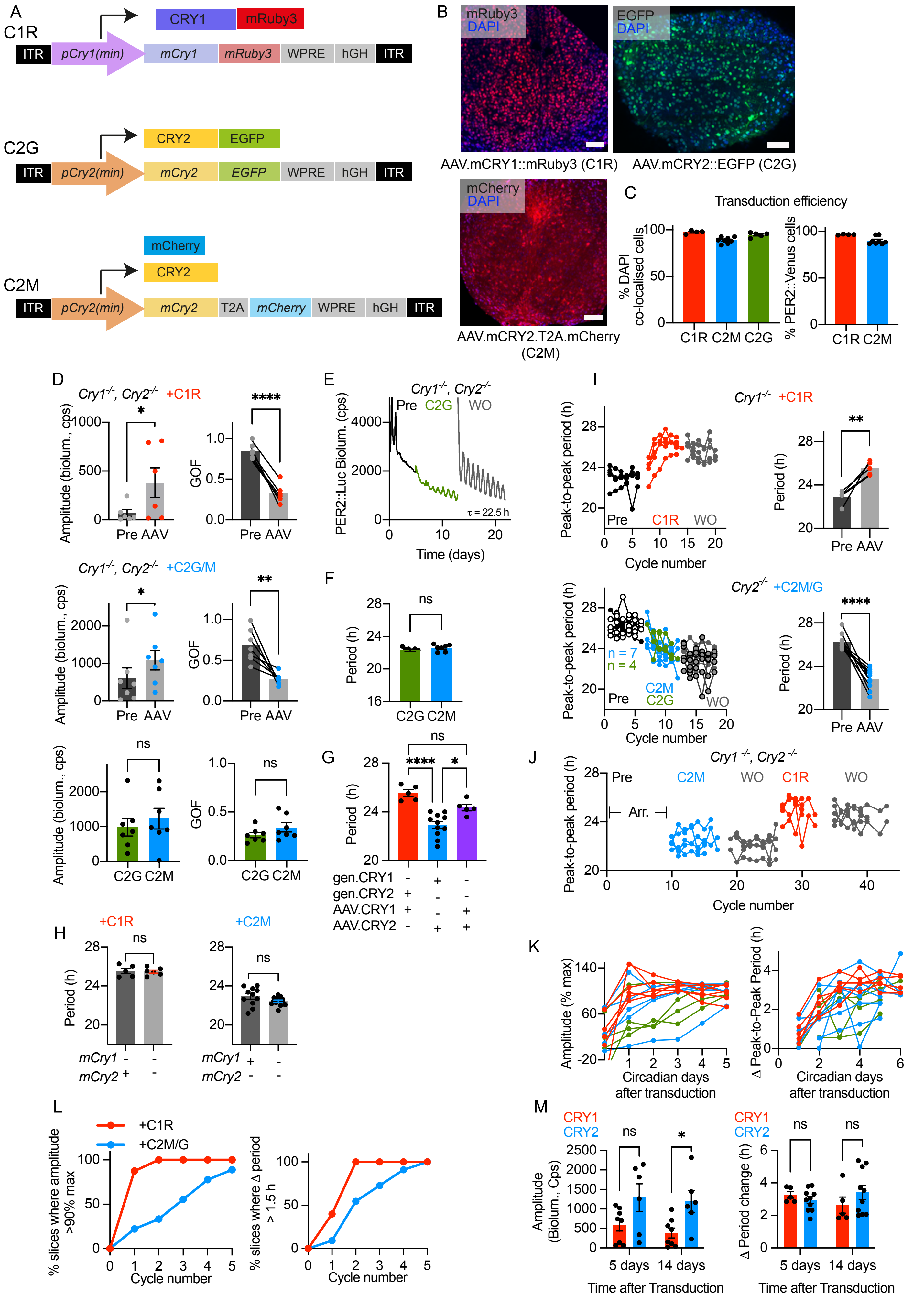

A
C1R
CRY1
mRuby3
ITR
pCry1(min)
mCry1
mRuby3
WPRE
hGH
ITR
C2G
CRY2
EGFP
ITR
pCry2(min)
mCry2
EGFP
WPRE
hGH
ITR
C2M
mCherry
CRY2
ITR
pCry2(min)
mCry2
T2A
mCherry
WPRE
hGH
ITR
B
mRuby3
DAPI
EGFP
DAPI
AAV.mCRY1::mRuby3 (C1R)
AAV.mCRY2::EGFP (C2G)
mCherry
DAPI
AAV.mCRY2.T2A.mCherry
(C2M)
C
Transduction efficiency
% DAPI co-localised cells
% PER2::Venus cells
C1R C2M C2G
C1R C2M
D
Cry1-/-, Cry2-/- +C1R
Amplitude (biolum., cps)
GOF
Pre AAV
Cry1-/-, Cry2-/- +C2G/M
E
Cry1-/-, Cry2-/-
Pre C2G WO
PER2::Luc Biolum. (cps)
τ = 22.5 h
Time (days)
F
Period (h)
C2G C2M
G
gen.CRY1 - + -
gen.CRY2 + - -
AAV.CRY1 + - +
AAV.CRY2 - + +
H
+C1R
+C2M
mCry1
mCry2
I
Cry1-/- +C1R
Peak-to-peak period (h)
Pre C1R WO
Cycle number
Cry2-/- +C2M/G
n = 7
n = 4
Pre C2M C2G WO
J
Cry1 -/-, Cry2 -/-
Pre C2M WO C1R WO
Arr.
K
Amplitude (% max)
Δ Peak-to-Peak Period (h)
Circadian days after transduction
L
+C1R
+C2M/G
% slices where amplitude >90% max
% slices where Δ period > 1.5 h
Cycle number
M
CRY1
CRY2
Amplitude (Biolum., Cps)
Δ Period change (h)
5 days 14 days
Time after Transduction

Figure S2. AAV-mediated expression reveals differential control by Cryptochromes 1 and 2 over SCN circadian rhythms.

(A) Schematic maps of AAV constructs and encoded CRY1 and CRY2 proteins. Note: C2M has a T2A linker resulting in the production of CRY2 and mCherry as separate proteins. (B) Representative confocal images of fixed SCN slices expressing (top left) C1R, (top right) C2G or (bottom left) C2M, counterstained with DAPI (blue). Scale bar = 100 μm. (C) Transduction efficiency of the 3 AAVs. Left: percentage of all SCN cells (co-localising AAV fluorescence with DAPI cell marker). Right: percentage of PER2+ cells (co-localising AAV fluorescence with P2V). (D) Group data comparing amplitude (left) and GOF (proxy for rhythm robustness) (right) of PER2::Luc rhythms before and after transduction of CryDKO SCN slices. Upper: C1R; Middle: Pooled C2G and C2M; Lower: C2G and C2M datasets separated. Connected dots indicate paired analyses (paired t-test). The remaining data were analysed by unpaired t-test. (E) Representative PER2::Luc bioluminescence trace for CryDKO SCN slice transduced with C2G. (F) Group data for circadian period of PER2::Luc rhythms in CryDKO SCN slices initiated by C2G or C2M (unpaired t-test; $p > 0.05$ ns). (G) Period analysis comparing different permutations of genomic or AAV-mediated CRY1 and 2 expression (one-way ANOVA: $p > 0.05$ ns, $p < 0.05$ *, $p < 0.0001$ ****). (H) Comparison of period of PER2::Luc rhythms for SCN from different *Cry* genetic backgrounds transduced with C1R (left) or C2M/G (right) (unpaired t-test; $p > 0.05$ ns). (I) Left, individual peak-to-peak period (P2P) and right, initial and final periods for C1R-transduced (upper) or C2M/G-transduced (lower) SCN slices. (J) P2P traces for individual serially transduced SCN slices. (K) Initiation dynamics of individual SCN slices. Left: percentage amplitude, right: change of P2P period. Note: mean±SEM data for (I-K) shown in Figure 2. (L) Initiation dynamics expressed as percentage of transduced CryDKO SCN slices that have reached >90% of their maximal amplitude (left) or percentage of transduced (red) Cry1KO and (blue) Cry2KO SCN slices that have exceeded a period change of 1.5 h (right). (M) Comparisons at 5 days and 14 days after transduction of initiated amplitude (left) and change in circadian period (right) in slices transduced by C1R (red) or C2M/G (blue) (2-way ANOVA with Šídák's multiple comparisons test; $p > 0.05$ ns, $p < 0.05$ *). For group data, dots represent individual SCN slices. Related to Figure 2.

Fig S3

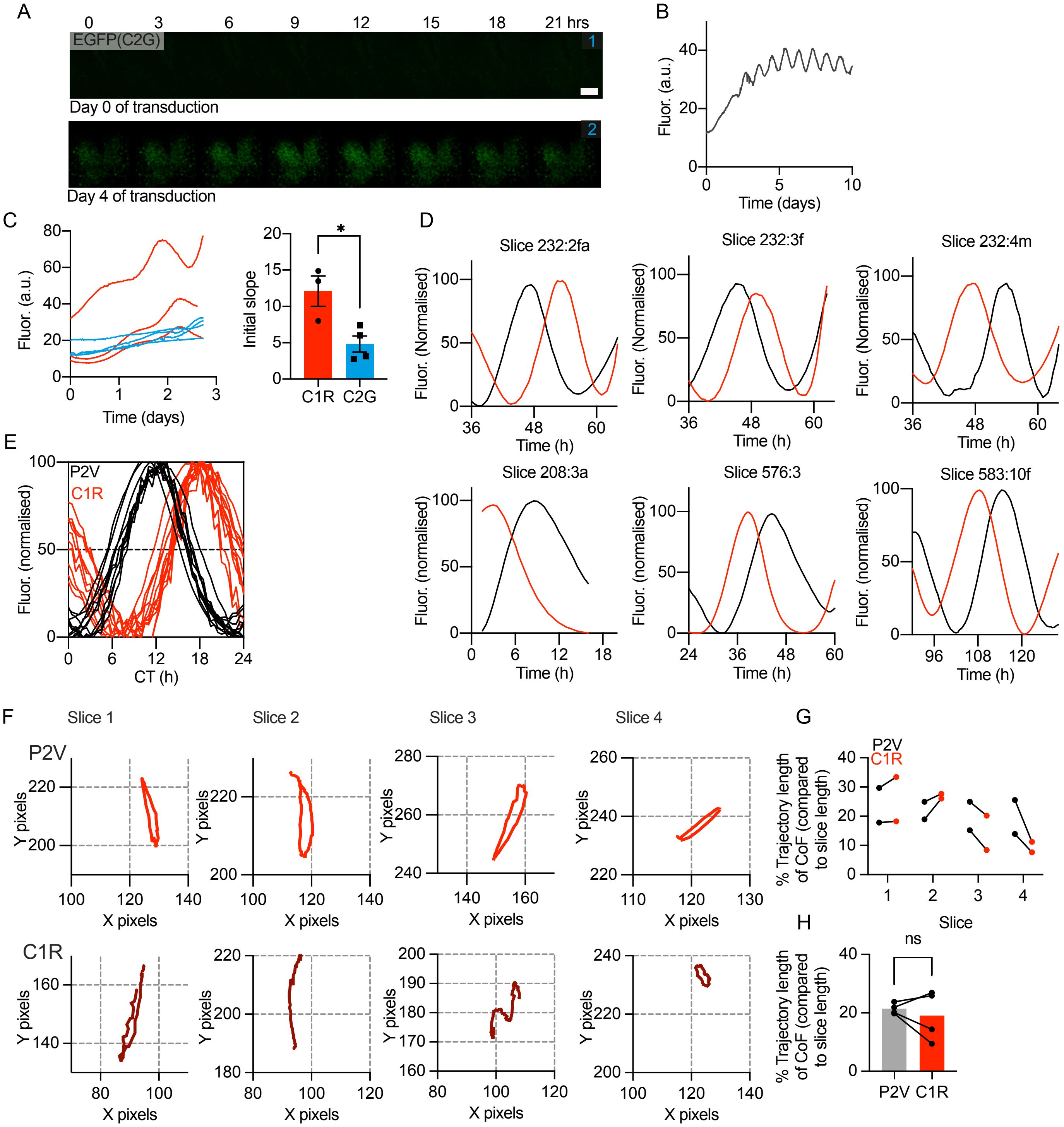
A
0 3 6 9 12 15 18 21 hrs
EGFP(C2G)
1
Day 0 of transduction
2
Day 4 of transduction
B
Fluor. (a.u.)
Time (days)
C
Fluor. (a.u.)
Time (days)
Initial slope
C1R C2G
D
Slice 232:2fa
Slice 232:3f
Slice 232:4m
Fluor. (Normalised)
Time (h)
Slice 208:3a
Slice 576:3
Slice 583:10f
Fluor. (normalised)
E
P2V
C1R
Fluor. (normalised)
CT (h)
F
Slice 1
Slice 2
Slice 3
Slice 4
P2V
C1R
Y pixels
X pixels
G
P2V
C1R
% Trajectory length of CoF (compared to slice length)
Slice
H
ns
% Trajectory length of CoF (compared to slice length)
P2V C1R

Figure S3. Dual-fluorescence time-lapse recordings of endogenous PER2::Venus and AAV-CRY1 or AAV-CRY2 in SCN slices.

(A) 24 h image montage from a confocal time-lapse recording of an SCN slice transduced with C2G. Upper: day 1 after transduction. Lower: day 4 after transduction. (B) Fluorescence intensity trace for C2G from confocal time-lapse recording shown in (A). (C) Left: fluorescence traces showing the first 60 hours of confocal time-lapse recordings of C1R (red) and C2G (blue) in transduced CryDKO SCN slices, demonstrate differences in initial expression dynamics. Right: the initial slope of expression of C1R and C2G fluorescence, as determined by linear regression analysis for the two proteins (t-test; $p < 0.05$ *). (D) Dual-fluorescence traces for endogenous P2V and AAV-expressed C1R in individual SCN. (E) Aligned fluorescence profiles (P2V peaks aligned to CT12) for individual slices used to generate the mean profiles shown in Figure 2F. (F) Four pairs of representative CoF plots for P2V (upper) and C1R (lower) from individual slices. Note: P2V CoF for slice 1 is replotted from Fig. 2H. (G) % trajectory lengths for P2V and C1R from slices shown in (F). Two trajectories per reporter are measured for each slice: one for each bilateral SCN. (H) Paired comparison of mean ± SEM % trajectory length for P2V and C1R within the same slices (Paired t-test; $p > 0.05$ ns). Related to Figure 2.

Fig S4

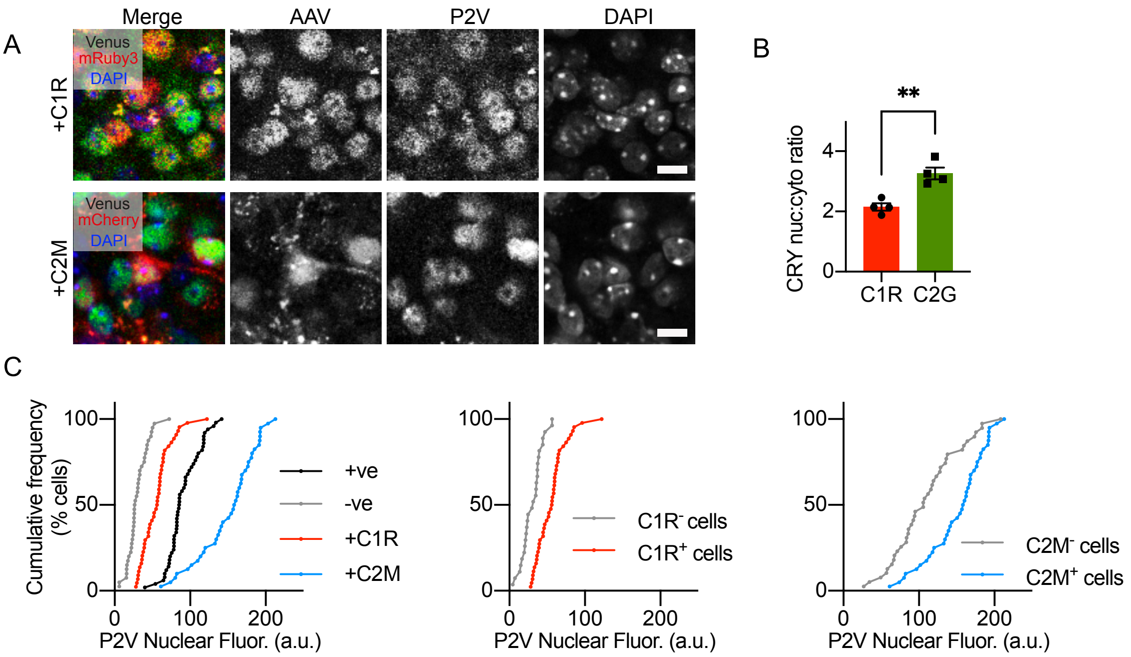

A
Merge
AAV
P2V
DAPI
+C1R
Venus
mRuby3
DAPI
+C2M
Venus
mCherry
DAPI
B
CRY nuc:cyto ratio
**
0
2
4
C1R
C2G
C
Cumulative frequency (% cells)
100
50
0
P2V Nuclear Fluor. (a.u.)
0
100
200
+ve
-ve
+C1R
+C2M
C1R- cells
C1R+ cells
C2M- cells
C2M+ cells

Figure S4. Intracellular localisation of P2V and AAV-driven CRY fusions.

(A) Representative confocal images showing intracellular localisation of P2V (green) in cells from SCN slices transduced with (upper) C1R or (lower) C2M (both shown in red), co-stained with DAPI nuclear marker (blue). Channels also shown separately as greyscale. Scale bar = 10 μm. (B) Nuc:Cyto ratios for C1R and C2G (t-test; $p < 0.001$ **). Note: C2G was used rather than C2M as C2M releases free mCherry rather than being a CRY2 fusion. (C) Cumulative frequency graphs showing nuclear P2V fluorescence intensity measured in SCN cells across multiple slices ($n > 26$ cells per group, $N > 3$ slices per group). Left: Comparison between slices (+ve = WT; -ve = CryDKO). Middle and Right: Comparison of un-transduced (grey) and transduced cells (red or blue) within CryDKO SCN slices transduced with (middle) C1R or (right) C2M. Related to Figure 3.

Fig S5

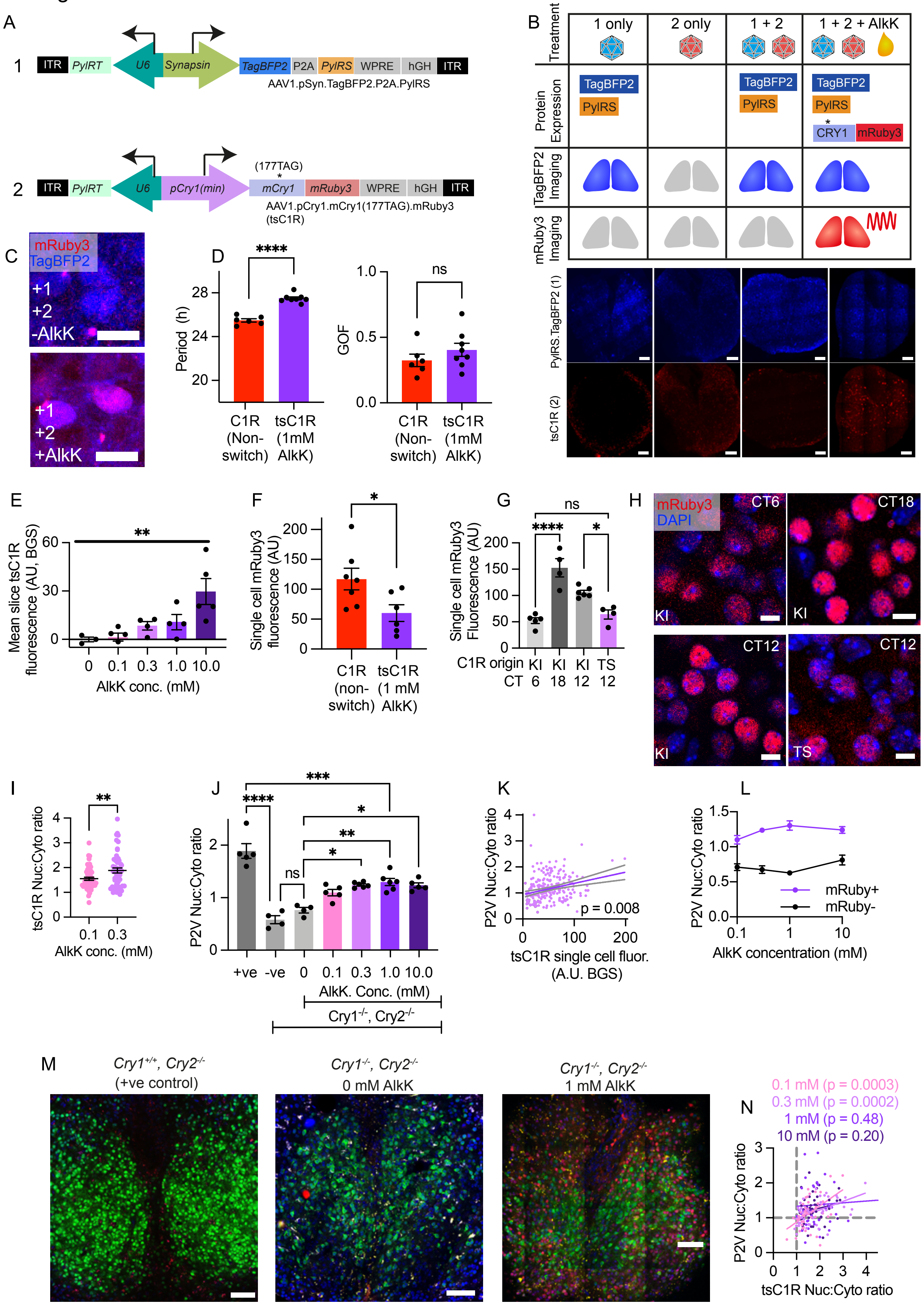

A
1
ITR
PylRT
U6
Synapsin
TagBFP2
P2A
PylRS
WPRE
hGH
ITR
AAV1.pSyn.TagBFP2.P2A.PylRS
2
ITR
PylRT
U6
pCry1(min)
(177TAG)
mCry1
mRuby3
WPRE
hGH
ITR
AAV1.pCry1.mCry1(177TAG).mRuby3
(tsC1R)
B
Treatment
1 only
2 only
1 + 2
1 + 2 + AlkK
Protein Expression
TagBFP2
PylRS
CRY1
mRuby3
TagBFP2 Imaging
mRuby3 Imaging
PylRS.TagBFP2 (1)
tsC1R (2)
C
mRuby3
TagBFP2
+1
+2
-AlkK
+AlkK
D
Period (h)
C1R (Non-switch)
tsC1R (1mM AlkK)
GOF
ns
E
Mean slice tsC1R fluorescence (AU, BGS)
AlkK conc. (mM)
F
Single cell mRuby3 fluorescence (AU)
C1R (non-switch)
tsC1R (1 mM AlkK)
G
Single cell mRuby3 Fluorescence (AU)
C1R origin
CT
KI
TS
H
mRuby3
DAPI
CT6
CT18
CT12
CT12
KI
TS
I
tsC1R Nuc:Cyto ratio
AlkK conc. (mM)
J
P2V Nuc:Cyto ratio
+ve
-ve
AlkK. Conc. (mM)
Cry1-/-, Cry2-/-
K
P2V Nuc:Cyto ratio
p = 0.008
tsC1R single cell fluor. (A.U. BGS)
L
P2V Nuc:Cyto ratio
mRuby+
mRuby-
AlkK concentration (mM)
M
Cry1+/+, Cry2-/- (+ve control)
Cry1-/-, Cry2-/- 0 mM AlkK
Cry1-/-, Cry2-/- 1 mM AlkK
N
0.1 mM (p = 0.0003)
0.3 mM (p = 0.0002)
1 mM (p = 0.48)
10 mM (p = 0.20)
P2V Nuc:Cyto ratio
tsC1R Nuc:Cyto ratio

Figure S5. Translationally switchable CRY1 reveals dose dependent control of PER2 localisation.

(A) Schematic showing constructs used for dual-AAV translational switching system. Note that codon encoding amino acid 177 of CRY1 is mutated to a TAG amber stop codon, thus enabling translational control by provision of AlkK. (B) Schematic table with (below) accompanying representative confocal images showing control experiments. Both AAVs plus application of the non-canonical amino acid (AlkK) are necessary for expression of tsC1R. Scale bar = 100 μm. (C) Close-up confocal images of fixed SCN slices transduced with both AAVs required for tsC1R expression. Expression of tsC1R does not occur in the absence of AlkK (left), whereas tsC1R expression does occur in the presence of AlkK (right). Scale bar = 10 μm. (D) Comparison (unpaired t-test) of properties of initiated PER2::Luc rhythms in CryDKO SCN slices expressing either non-switchable C1R (red) or translationally switched tsC1R (purple). Left: Circadian Period. Right: Goodness-of-Fit (GOF). (E) Background subtracted (BGS) mean slice fluorescence intensity of tsC1R across SCN slices treated with different concentrations of AlkK (One-way ANOVA, significant dose dependent effect: $p < 0.001$ **). (F) mRuby3 whole-cell fluorescence intensity measured as a proxy for [CRY1] in non-switched C1R and tsC1R (1mM AlkK) in SCN slices, fixed at CT12 (unpaired t-test). (G) Measure of the functional threshold of CRY1 relative to endogenous levels. As in (F), but instead comparing tsC1R +0.3 mM AlkK (TS) with endogenous C1R knock-in (KI) measured at CT12, the same timepoint as for tsC1R, as well as at the nadir (CT6) and peak (CT18) of CRY1 expression (One-way ANOVA). (H) Representative confocal images showing (left) endogenous (KI) and (right) translationally switched (with 0.3mM AlkK) (TS) CRY1::mRuby3 expression in SCN slices. Scale bar = 10 µm. (I) Single-cell measures showing that a significantly higher tsC1R Nuc:Cyto ratio in slices treated with 0.3 mM AlkK compared with 0.1 mM AlkK (unpaired t-test). (J) Group data for P2V Nuc:Cyto ratios across increasing doses of AlkK in tsC1R-transduced slices, compared with WT (+ve) and CryDKO (untransduced, -ve) slices (one-way ANOVA). Note that at the AlkK concentrations tested, P2V Nuc:Cyto does not reach WT levels. (K) Pearson's correlation showing positive correlation between single cell tsC1R fluorescence intensity and P2V Nuc:Cyto ratio. The trend line is bound by 95% confidence limits (grey line). (L) Comparison of P2V Nuc:Cyto ratio between un-transduced (black) and transduced (purple) cells within SCN slices transduced with tsC1R and treated with increasing doses of AlkK. (M) Representative confocal images showing P2V (green) and tsC1R (red) expression and counterstained with DAPI (blue). Scale bar = 100 μm. (N) Pearson's correlation and linear regression line fits between tsC1R Nuc:Cyto ratio and, P2V Nuc:Cyto ratio, in SCN slices treated with different AlkK concentrations. Note that positive correlation collapses with saturating effect of 1mM AlkK. For all group data, bars represent the mean and error bars represent SEM. The dots represent individual slices in (D-F and G), the mean value across slices in (L), and individual cells in (I, K and N). Note cells originate from multiple SCN slices in I, K and N. For all comparative statistics: $p > 0.05$ ns, $p < 0.05$ *, $p < 0.01$ **, $p < 0.001$ ***, $p < 0.0001$ ****. Related to Figure 3.

Fig S6

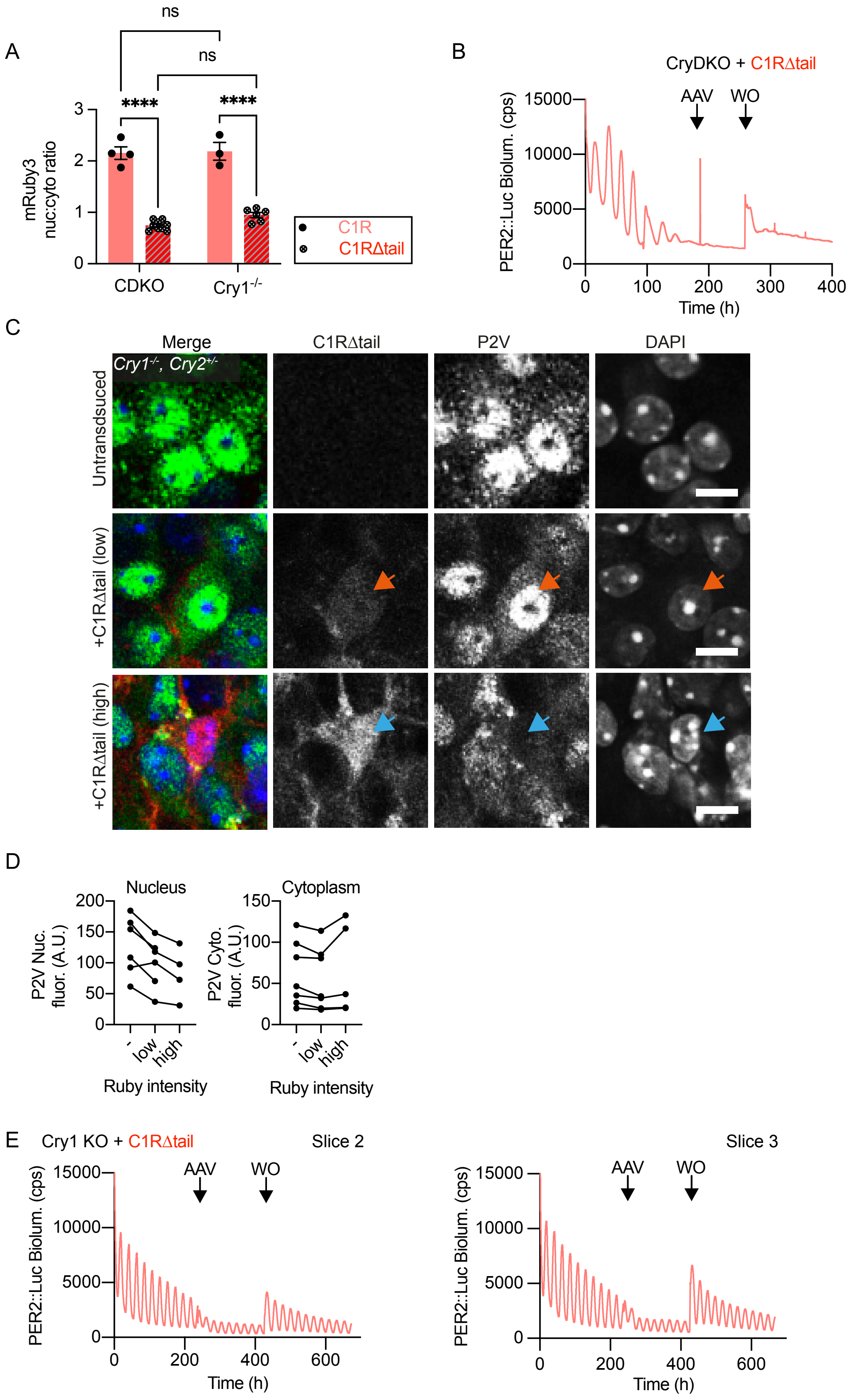
A
ns
ns
****
****
mRuby3 nuc:cyto ratio
CDKO
Cry1-/-
C1R
C1RΔtail
B
CryDKO + C1RΔtail
AAV
WO
PER2::Luc Biolum. (cps)
Time (h)
C
Merge
C1RΔtail
P2V
DAPI
Cry1-/-, Cry2+/-
Untransdsuced
+C1RΔtail (low)
+C1RΔtail (high)
D
Nucleus
Cytoplasm
P2V Nuc. fluor. (A.U.)
P2V Cyto. fluor. (A.U.)
- low high
Ruby intensity
E
Cry1 KO + C1RΔtail
Slice 2
Slice 3
AAV
WO
PER2::Luc Biolum. (cps)
Time (h)

Figure S6. The C-terminal domain (CTD) of CRY1 is necessary for initiation of circadian oscillation and normal period-setting in the SCN, and for nuclear localisation of PER2.

(A) Nuc:Cyto ratios of C1R in cells from either CryDKO or Cry1KO SCN slices transduced with either full-length C1R or truncated C1RΔtail (two-way ANOVA with Tukey's multiple comparisons test, $p > 0.05$ ns, $p < 0.0001$ ****). (B) Representative PER2::Luc bioluminescence trace of a CryDKO SCN slice, showing that transduction with AAV-C1RΔtail did not initiate circadian rhythmicity. (C) Representative confocal images showing variable intracellular localisation of P2V (green) in Cry1KO SCN slices transduced with C1RΔtail (red), co-stained with DAPI (blue). The threshold for "low" versus "high" levels of C1RΔtail was 100 fluorescence AU of mRuby tag (using same imaging settings across all measurements). Red arrows highlight a cell expressing low levels of C1RΔtail, with nuclear P2V signal but also noticeable levels of cytoplasmic P2V. The blue arrows highlight a cell expressing high levels of C1RΔtail, with corresponding very low levels of nuclear P2V and diffuse cytoplasmic signal. Scale bar = 10 μm. (D) Intensity of P2V fluorescence in nucleus (left) and cytoplasm (right) of Cry1KO SCN cells exhibiting zero, low or high expression of C1RΔtail, as exemplified in (C). Each set of connected dots represents mean cell values for individual SCN slices. Note that not all categories are represented in every SCN slice. (E) Representative PER2::Luc traces for Cry1KO SCN slices, transduced as in (C). Related to Figure 4.

Fig S7

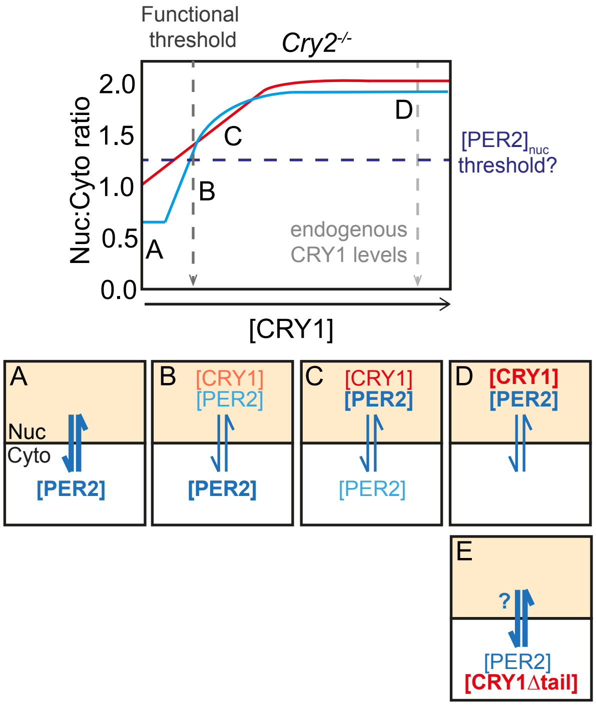
Functional threshold
Cry2-/-
2.0
1.5
1.0
0.5
0.0
Nuc:Cyto ratio
A
B
C
D
[PER2]nuc threshold?
endogenous CRY1 levels
[CRY1]
A
Nuc
Cyto
[PER2]
B [CRY1] [PER2]
[PER2]
C [CRY1] [PER2]
[PER2]
D [CRY1] [PER2]
E
?
[PER2]
[CRY1Δtail]

Figure S7. The relationship between CRY1 expression and PER2 intracellular behaviour Upper: Illustrative graph showing localisation (Nuc:Cyto ratio) of PER2 (blue) and CRY1 (red) with respect to CRY1 protein expression. Dark grey dashed line: threshold of CRY1 expression that is sufficient to support SCN-wide circadian rhythms. Dark blue dashed line: proposed threshold for nuclear-localised PER2 sufficient to maintain SCN rhythmicity, based on the PER2 nuc:cyto ratio measured at CRY1 threshold. Light grey dashed line: the endogenous expression of CRY1 (S5G-H), which is well above the functional threshold. Lower: Schematic showing mobility and localisation of PER2 and CRY1 at different levels of CRY1 expression. Each panel corresponds to a different level of CRY1 expression with respect to the threshold, as labelled in the upper graph: A) No CRYs; B) below CRY1 threshold; C) above CRY1 threshold; D) at endogenous CRY1 levels. E) illustrates the effect of the CRY1 C-terminal tail. Without its tail, it cannot maintain nuclear retention of PER2. Given that the tail would normally form interactions with PER2, we propose that PER2 mobility would be increased compared to WT. Related to Figures 3 and 4.

Supplementary Videos:

Supplementary videos show representative confocal recordings of FRAP experiments. To minimise the effect of acquisition bleaching, and optimise for acquisition speed, only the ROIs were acquired, thus these are not full-frame videos. Frame rate = 2fps.

Video S1.

Whole nucleus FRAP of WT SCN slice at CT12.

Video S2.

Whole cytoplasm FRAP of WT SCN slice at CT12.

Video S3.

Whole nucleus FRAP of CryDKO SCN slice (CT not applicable).

Video S4.

Whole cytoplasm FRAP of CryDKO SCN slice at (CT not applicable).

References

1. G. T. J. van der Horst *et al.*, Mammalian Cry1 and Cry2 are essential for maintenance of circadian rhythms. *Nature* **398**, 627-630 (1999).
2. S. H. Yoo *et al.*, PERIOD2 :: LUCIFERASE real-time reporting of circadian dynamics reveals persistent circadian oscillations in mouse peripheral tissues. *P Natl Acad Sci USA* **101**, 5339-5346 (2004).
3. E. S. Maywood *et al.*, Analysis of core circadian feedback loop in suprachiasmatic nucleus of mCry1-luc transgenic reporter mouse. *P Natl Acad Sci USA* **110**, 9547-9552 (2013).
4. N. J. Smyllie *et al.*, Visualizing and Quantifying Intracellular Behavior and Abundance of the Core Circadian Clock Protein PERIOD2. *Current biology : CB* **26**, 1880-1886 (2016).
5. J. S. Bagnall *et al.*, Quantification of circadian interactions and protein abundance defines a mechanism for operational stability of the circadian clock. *BioRXiV* **[Preprint]** (2021).
6. M. D. Edwards, M. Brancaccio, J. E. Chesham, E. S. Maywood, M. H. Hastings, Rhythmic expression of cryptochrome induces the circadian clock of arrhythmic suprachiasmatic nuclei through arginine vasopressin signaling. *P Natl Acad Sci USA* **113**, 2732-2737 (2016).
7. R. J. Ernst *et al.*, Genetic code expansion in the mouse brain. *Nat Chem Biol* **12**, 776-+ (2016).
8. E. S. Maywood *et al.*, Translational switching of Cry1 protein expression confers reversible control of circadian behavior in arrhythmic Cry-deficient mice. *Proc Natl Acad Sci U S A* **115**, E12388-E12397 (2018).
9. M. H. Hastings, A. B. Reddy, D. G. McMahon, E. S. Maywood, Analysis of circadian mechanisms in the suprachiasmatic nucleus by transgenesis and biolistic transfection. *Method Enzymol* **393**, 579-592 (2005).
10. M. Brancaccio, E. S. Maywood, J. E. Chesham, A. S. I. Loudon, M. H. Hastings, A Gq-Ca2+ Axis Controls Circuit-Level Encoding of Circadian Time in the Suprachiasmatic Nucleus. *Neuron* **78**, 714-728 (2013).
11. D. Axelrod, D. E. Koppel, J. Schlessinger, E. Elson, W. W. Webb, Mobility Measurement by Analysis of Fluorescence Photobleaching Recovery Kinetics. *Biophys J* **16**, 1055-1069 (1976).
12. J. Schindelin *et al.*, Fiji: an open-source platform for biological-image analysis. *Nature Methods* **9**, 676-682 (2012).
13. T. Zielinski, A. M. Moore, E. Troup, K. J. Halliday, A. J. Millar, Strengths and Limitations of Period Estimation Methods for Circadian Data. *Plos One* **9** (2014).
14. M. M. Dorostkar, E. Dreosti, B. Odermatt, L. Lagnado, Computational processing of optical measurements of neuronal and synaptic activity in networks. *J Neurosci Meth* **188**, 141-150 (2010).
